# Graph-based contrastive learning enables unified integration and niche transfer across single-cell and spatial multi-omics

**DOI:** 10.1093/bib/bbag432

**Published:** 2026-08-18

**Authors:** Weige Zhou, Xueying Fan, Lanxiang Li, Jianrong Zheng, Xiaodong Liu, Wenfei Jin, Luyi Tian

**Affiliations:** GMU-GIBH Joint School of Life Sciences, Guangzhou Medical University, No. 1 Xinzao Road, Xinzao Town, Panyu District, Guangzhou, Guangdong 511436, China; Guangzhou National Laboratory, No. 9 Xingdao Huanbei Road, Guangzhou International Bio Island, Haizhu District, Guangzhou, Guangdong 510005, China; School of Life Sciences, Westlake University, No. 600 Dunyu Road, Xihu District, Hangzhou, Zhejiang 310030, China; CAS Key Laboratory of Computational Biology, Shanghai Institute of Nutrition and Health, Chinese Academy of Sciences, 320 Yueyang Road, Xuhui District, Shanghai 200031, China; GMU-GIBH Joint School of Life Sciences, Guangzhou Medical University, No. 1 Xinzao Road, Xinzao Town, Panyu District, Guangzhou, Guangdong 511436, China; Guangzhou National Laboratory, No. 9 Xingdao Huanbei Road, Guangzhou International Bio Island, Haizhu District, Guangzhou, Guangdong 510005, China; Guangzhou National Laboratory, No. 9 Xingdao Huanbei Road, Guangzhou International Bio Island, Haizhu District, Guangzhou, Guangdong 510005, China; CAS Key Laboratory of Computational Biology, Shanghai Institute of Nutrition and Health, Chinese Academy of Sciences, 320 Yueyang Road, Xuhui District, Shanghai 200031, China; School of Life Sciences, Westlake University, No. 600 Dunyu Road, Xihu District, Hangzhou, Zhejiang 310030, China; School of Life Sciences, Southern University of Science and Technology, Shenzhen 518055, China; GMU-GIBH Joint School of Life Sciences, Guangzhou Medical University, No. 1 Xinzao Road, Xinzao Town, Panyu District, Guangzhou, Guangdong 511436, China; Guangzhou National Laboratory, No. 9 Xingdao Huanbei Road, Guangzhou International Bio Island, Haizhu District, Guangzhou, Guangdong 510005, China

**Keywords:** spatial omics, tissue niches, VGAE, tumor microenvironment

## Abstract

The rapid growth of single-cell and spatial omics has outpaced computational methods capable of unifying these data into a cohesive framework for tissue atlas construction and cross-sample analysis. A critical bottleneck lies in the inability of existing tools to co-embed cells from diverse technologies—spanning transcriptomics, epigenomics, and proteomics—into a shared reference space while preserving spatial architecture and molecular specificity. Here, we present Garfield (Graph-based Contrastive Learning Enables Fast Single-Cell Embedding), a geometric deep-learning framework that addresses these challenges through spatially or molecularly aware cell embedding. Leveraging a graph contrastive learning framework, Garfield learns a shared embedding space for data generated by diverse technologies, enabling seamless construction and querying of spatial reference atlases. Our results show that Garfield consistently outperforms state-of-the-art benchmark models in identifying spatial niches across multiple datasets. We further demonstrate Garfield’s versatility by applying it to multimodal spatial data, including gene expression and chromatin accessibility, where it successfully identifies distinct niches in the mouse brain. Notably, Garfield reveals tumor microenvironment heterogeneity in non-small cell lung cancer and breast cancer, uncovered conserved, barrier-like immune niches at tumor margins orchestrating CD80-mediated T cell–B cell–dendritic cell interactions and IFN-*γ*/B cell activation pathways, forming spatially coordinated immune surveillance hubs. These findings underscore Garfield’s potential to advance spatial omics research by offering a robust, scalable solution for integrating and interpreting complex spatial data across diverse tissue types and modalities.

## Introduction

Recent advances in single-cell omics have enabled both individual and integrated cellular profiling. Single-cell multi-omics technologies now enable simultaneous measurements across multiple cellular layers, such as transcriptomics, epigenomics, and proteomics, significantly enhancing our understanding of cell states and the molecular mechanisms underlying development and disease. For example, CITE-seq [1] and REAP-seq [2] enable simultaneous detection of RNA expression and surface protein abundance in single cells, while SNARE-seq [3], SHARE-seq [4], 10x Multiome (https://www.10xgenomics.com/products/), and ISSAAC-seq [5] provide information on both RNA expression and chromatin accessibility from the same cell. Building on these advancements, spatial transcriptomics has emerged as a powerful approach that maps gene expression within the spatial context of tissues, adding a new layer of information. With the expansion to spatial multi-omics, researchers can now profile multiple omic layers (e.g. transcriptomics, epigenomics) within a single tissue section simultaneously, capturing complementary biological insights. Current spatial technologies are broadly divided into sequencing-based approaches [6–10], which infer spatial information by capturing and sequencing RNA, and imaging-based [11–13] approaches, which directly visualize RNA distribution using microscopy, each unlocking multidimensional, spatially resolved perspectives of each cell.

Despite their transformative potential, computational challenges persist in maximizing the utility of these technologies. Integration methods for single-cell multimodal data often depend on identifying ”anchors” to align datasets, either explicitly or implicitly. Based on anchor choice, integration approaches are typically categorized into horizontal, vertical, and diagonal integration [14]. We focus on state-of-the-art algorithms for horizontal and vertical integration. Horizontal integration, as in Seurat v3’s CCA [15], MultiVI [16], Multigrate [17], and MOFA+ [18], uses shared modalities to link datasets with differing cells. Conversely, vertical integration aligns datasets across omics layers, illustrated by tools like Seurat v4’s WNN [19] and MultiVI [16]. Spatial computational methods for identifying cell niches, such as GraphST [20], STAGATE [21], CellCharter [22], SPACE [23], and NicheCompass [24], have also been developed, yet these are designed solely for spatial transcriptomic data. Consequently, integrating or analyzing single-cell or spatial transcriptome data often requires multiple tools, increasing the user’s workload. Furthermore, current methods largely lack functionality to fully leverage relationships across cellular features and multi-modalities within a single cell. Few tools support joint analysis of single-cell and spatial multi-omics data while preserving both molecular specificity and spatial topology, a critical requirement for constructing comprehensive tissue atlases. More broadly, graph neural networks (GNNs) and contrastive objectives are increasingly used across bioinformatics, including drug–target interaction prediction [25], multilabel mRNA localization [26], RNA structure and sequence modeling [27, 28], and graph-guided combinatorial optimization [29]. In parallel, generative models for unpaired or incomplete omics [30] address the related but distinct challenge of learning coherent representations when not every molecular layer is observed. These developments motivate graph-based, contrastive, and variational frameworks, while missing-modality recovery remains a separate task that requires dedicated masking and validation protocols.

To address these limitations, we present Garfield (Graph-based Contrastive Learning enables Fast Single-Cell Embedding), a versatile embedding framework that unifies heterogeneous single-cell and spatial (multi-omics) datasets within a shared latent space, supporting a variety of analysis tasks. Unlike existing methods designed specifically for either single-cell or spatial data, Garfield simultaneously processes both data types, regardless of modality. Garfield constructs a unified observation graph whose nodes represent cells or spatial units and whose edges encode molecular or spatial relationships. By leveraging variational graph autoencoders (VGAE) [31] alongside a contrastive strategy based on singular value decomposition (SVD) and optional maximum mean discrepancy (MMD)-based alignment, Garfield embeds cells into a low-dimensional, batch-invariant biological space, enabling batch correction and multi-omics integration at the single-cell or spatial levels. Inspired by architectural surgery [32], Garfield further enables weight-constrained fine-tuning to map new datasets to single-cell or spatial reference atlases.

Garfield’s unified framework supports tasks including (i) dimensionality reduction; (ii) batch correction and multi-modal integration; (iii) niche discovery in spatial data; and (iv) QueryToReference mapping. Its adaptability across tasks is achieved by adjusting the input graph constructed from single-cell or spatial data. Extensive testing on multiple single-cell multi-omics, spatial uni-modal, and dual-omics datasets demonstrates Garfield’s robust performance, either surpassing or matching current state-of-the-art methods. Additionally, Garfield efficiently scales to large spatial datasets, enabling the construction of comprehensive atlases spanning nearly a million cells through mini-batch processing. Finally, we have developed a comprehensive, scalable Python package with a seamless workflow for data preprocessing, graph construction, graph embedding training with PyTorch, and post-analysis visualization. Compatible with popular single-cell (Scanpy) [33] and spatial tools (Squidpy) [34], Garfield provides detailed documentation and tutorials available at https://garfield-bio.readthedocs.io.

## Results

### Overview of Garfield

**Figure 1:**
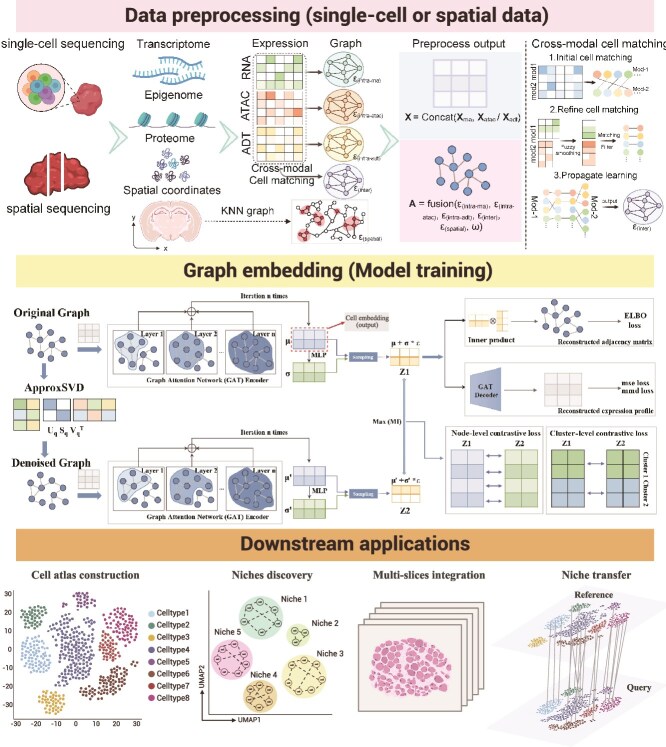
Garfield framework overview: Garfield takes single- or multi-modal single-cell and spatial omics data, including transcriptomic, epigenomic, proteomic, and spatial information, constructs molecular and spatial graphs, uses a graph neural network within a contrastive variational graph autoencoder framework to learn batch-invariant and spatially aware embeddings, and supports cell-atlas construction, niche discovery, multi-slice integration, and niche transfer; created with BioRender.com.

Garfield is a versatile embedding algorithm designed to support high-resolution analyses of single-cell or spatial data in both uni- and multi-modality formats (Fig. 1). By integrating multi-omics measurements with optional spatial information, Garfield characterizes molecular landscapes and spatial niches within tissue samples when spatial coordinates are available. Its flexible input includes feature matrices of segmented cells or capture locations (such as beads, pixels, bins, or spots), along with spatial coordinates. Because the modeled units can be cells, segmented subcellular objects, beads, bins, or spots depending on the platform, we use “observation” as the generic term and “spatial unit” for spatial assays. We reserve “cell” for single-cell-resolution measurements or for contexts where the platform resolution is unambiguous, consistent with the notation $N_{\mathrm{obs}}$ used throughout the Methods.

Garfield employs a VGAE [31] framework to embed observations within a spatially and molecularly coherent latent space using a self-supervised, multi-task learning approach. For single-cell data, Garfield constructs a molecular neighborhood graph, with nodes representing cells and edges indicating molecular similarity. For spatial data, it builds a spatial neighborhood graph using cell or spot coordinates, where edges capture spatial relationships between spatial units. Each node encodes a feature vector representing omics data, such as gene expression in unimodal analyses or paired gene expression with either chromatin accessibility peaks or antibodies in multimodal setups. Unlike previous methods [23, 35], which rely on single graph type (either molecular graph or spatial graph), Garfield concurrently integrates intra-modality molecular graph, inter-modality molecular graph, and spatial graph if available (derived from spatial coordinates).

To achieve spatially and molecularly integrated latent representations, Garfield offers several GNN encoders (GATv2 [36], GAT [37], and GCN [38]). To enhance biological signal while minimizing noise, Garfield incorporates a sparse approximation of Singular Value Decomposition (approxSVD) contrastive learning [39] strategy, maximizing mutual information (MI) between original and denoised node views. Unlike traditional variational autoencoders, which rely on the encoder to estimate both data mean and variance, Garfield’s encoder learns only the mean, with the variance calculated based on this mean through an additional fully connected layer. This adaptive variance scaling adjusts contrast loss per node, inherently reflecting biological variation across cells. Additionally, to mitigate batch effects, Garfield applies MMD [40] loss as an alignment strategy, which is widely utilized in batch correction [32, 41].

In the decoding phase, Garfield reconstructs both spatial and molecular information via multiple modules. A graph decoder aligns sample-specific latent representations to reconstruct spatial graphs through edge reconstruction loss, preserving spatial structure by enforcing similarity between neighboring nodes. For molecular information, Garfield utilizes an omics decoder composed of an inner product module and a feature reconstruction module to optimize omics feature recovery.

In essence, Garfield’s comprehensive framework is an expanded multimodal graph variational autoencoder. This design enables precise characterization of single-cell landscapes and tissue-specific niches, offering a versatile, end-to-end framework for single-cell and spatial omics data analysis.

### Benchmarking Garfield for single-cell multi-omics integration

**Figure 2:**
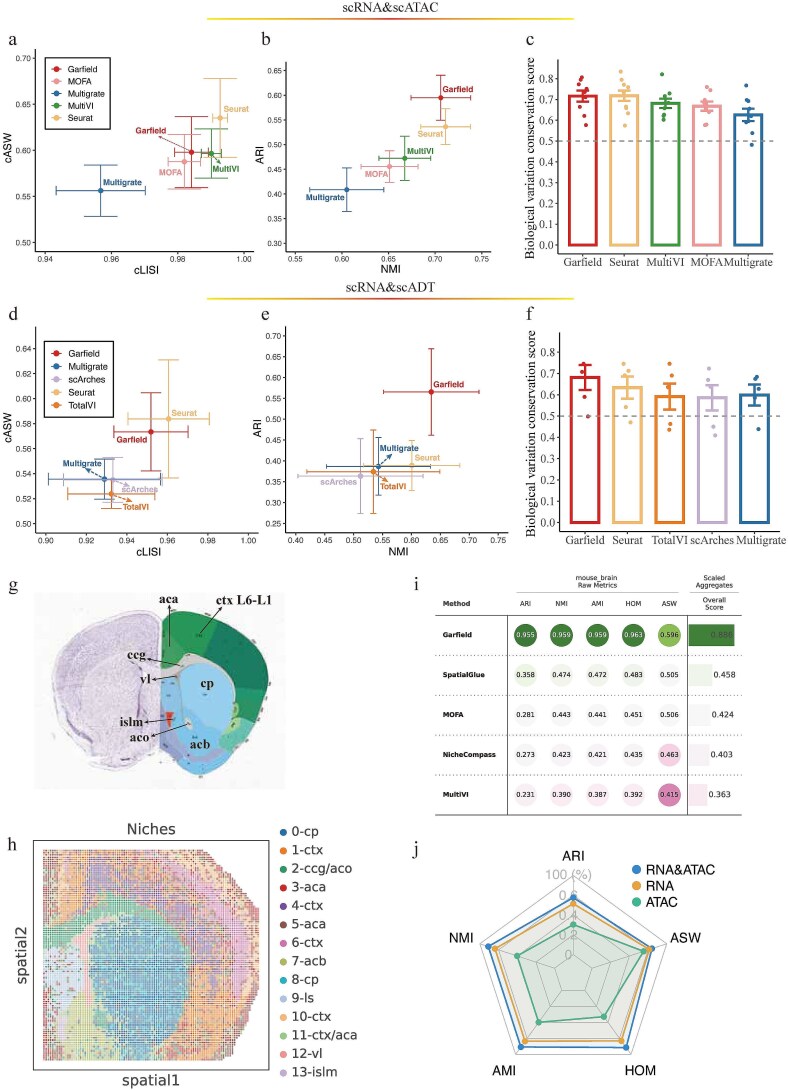
Garfield outperforms existing state-of-the-art methods in single-cell and spatial multimodal integration: (a–c) cLISI, cASW, NMI, ARI, and biological variation conservation scores compare five integration methods across nine paired single-cell RNA and ATAC datasets, with data in a and b presented as mean *±* standard error and data in c as means with 95% confidence intervals (*N = 9* datasets); (d–f) the corresponding analyses compare five methods across five paired single-cell RNA and protein datasets, with data presented as means with 95% confidence intervals (*N = 5* datasets); (g,h) an annotated coronal section from the Allen Mouse Brain Atlas and the corresponding Garfield-derived spatial niches show the caudoputamen (cp), cerebral cortex (ctx), genu of corpus callosum (ccg), anterior commissure, olfactory limb (aco), anterior cingulate area (aca), nucleus accumbens (acb), lateral septal nucleus (ls), lateral ventricle (vl), lateral preoptic area (lpo), and major island of Calleja (islm); (i) ARI, NMI, AMI, HOM, and ASW compare Garfield, SpatialGlue, MOFA+, NicheCompass, and MultiVI; and (j) the same metrics compare RNA, ATAC, and joint RNA–ATAC representations.

Single-cell multi-omics technologies enable researchers to integrate genomic information, providing insights into cellular functions [19], gene regulation [42], and precise fate prediction [43, 44]. To evaluate Garfield’s performance in single-cell multi-omics integration, we compared it with four widely used algorithms—Seurat V4, MultiVI, MOFA+, and Multigrate—on nine scRNA + scATAC datasets (Fig. 2a–c, Supplementary Table S1). Clustering was performed using the Leiden algorithm, matching the number of clusters to ground truth cell types. Performance was assessed with four metrics: adjusted Rand index (ARI), normalized mutual information (NMI), cell-type average silhouette width (cASW), and cell-type separation local inverse Simpson’s index (cLISI). The biological variation conservation score, calculated as the average of these metrics, was used as an overall performance indicator.

Garfield achieved the highest scores, including an average ARI of 0.59, NMI of 0.71, cASW of 0.59, and cLISI of 0.98 (Fig. 2a and b). Seurat V4 also performed well with ARI of 0.53, NMI of 0.71, cASW of 0.63, and cLISI of 0.99, resulting in biological variation conservation scores of 0.716 and 0.718 for Garfield and Seurat V4, respectively, outperforming MultiVI (0.681), Multigrate (0.626), and MOFA+ (0.668; Fig. 2c). Garfield was further evaluated alongside Seurat V4, TotalVI, scArches, and Multigrate on five scRNA + scADT datasets (Supplementary Table S2). Garfield displayed the highest average ARI (0.57), NMI (0.63; Fig. 2e), and superior biological variation conservation (Garfield: 0.68; Seurat V4: 0.63; TotalVI: 0.59; scArches: 0.59; Multigrate: 0.60; Fig. 2f). The detailed performance metrics of various algorithms across different datasets are presented in Fig. S1. For clarity, we highlight one representative dataset that showcases Garfield’s ability to capture cross-modality representations from parallel single-cell RNA–ATAC or RNA–ADT datasets (Figs S2 and S3).

Overall, comprehensive benchmarking shows Garfield outperforms or matches current mainstream tools in integrating single-cell multi-omics (scRNA + scATAC and scRNA + scADT).

### Benchmarking Garfield for spatial multi-omics integration

Spatial transcriptomics represents the next frontier in biological sample analysis following the success of single-cell transcriptomics [45]. With advancements in spatial technologies, it is now possible to profile multiple omics layers simultaneously on a single tissue section [46]. Notably, Garfield is also suitable for integrating spatial multi-omics data.

To evaluate Garfield’s capabilities, we benchmarked it against competing methods—NicheCompass, SpatialGlue, MultiVI, and MOFA+—using a mouse brain epigenome–transcriptome dataset. The Allen Brain Atlas reference was used to annotate anatomical regions such as the anterior cingulate area (aca), cortex layers (ctx), genu of corpus callosum (ccg), lateral septal nucleus (ls), major island of Calleja (islm), and nucleus accumbens (acb; Fig. 2g and h). Distinct spatial distributions were observed for each anatomical region, with clear conformity between subcomponents (Fig. S4a and b).

Benchmarking involved applying Leiden clustering across various resolutions on the integrated results from each method to minimize bias from resolution parameters (Methods). Performance in spatial niche detection was assessed using five metrics: ARI, NMI, adjusted mutual information (AMI), homogeneity (Homo), and niche average silhouette width (ASW). Garfield outperformed competitors, achieving improvements of 59.7%, 48.5%, 48.7%, 48.0%, and 9.0% over the second-best method across these metrics, with an average improvement of 42.78% (Fig. 2i).

Qualitative evaluation through Uniform Manifold Approximation and Projection (UMAP) and spatial visualizations demonstrated Garfield’s superior ability to identify spatial niches (Fig. S4c–e). While single-cell multi-omics methods like MultiVI and MOFA+ struggled to distinguish niches, and spatial multi-omics methods like NicheCompass and SpatialGlue showed limited performance in fine tissue niches (e.g. islm), Garfield effectively resolved detailed niche structures, including ctx layers, ccg/aco, ls, acb, and vl. Importantly, Garfield uniquely identified the sophisticated niche structure of islm, which competing algorithms failed to detect. Moreover, our findings indicate that Garfield surpasses single-modal approaches by effectively integrating multi-modal information, thereby enhancing the quality of representation learning (Fig. 2j).

Additionally, Garfield exhibited robust performance across varying neighbor counts and random seeds (Fig. S5). These results underscore Garfield’s potential in cross-modality representation, spatial niche identification, and downstream visualization, establishing it as a powerful tool for spatial multi-omics integration.

### Garfield outperforms state-of-the-art tools for tissue niche detection

To evaluate Garfield’s performance in tissue niche identification, we compared it with other spatial representation methods, including NicheCompass [24], CellCharter [22], and GraphST [20], focusing on cellular representations and niche labels. The benchmarking was conducted on a Slide-seqV2 dataset of the mouse hippocampus, known for its well-defined spatial tissue organization that closely aligns with anatomical structures [6].

**Figure 3:**
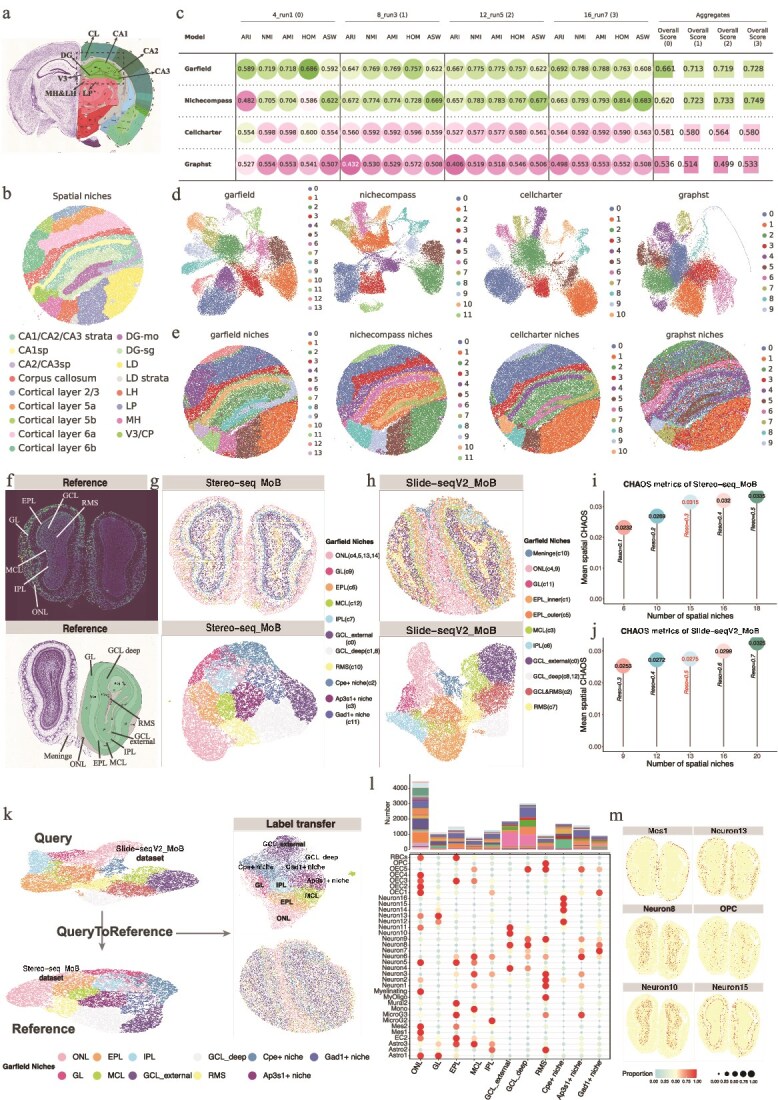
Garfield accurately identifies niches across technologies and tissues: (a,b) anatomical annotations from the Allen Mouse Brain Reference Atlas and manually defined hippocampal niches represent the corpus callosum, lateral posterior thalamic nucleus, stratum, medial and lateral habenula, stria medullaris, dentate gyrus, CA1, CA2, CA3, and third ventricle; (c–e) ARI, NMI, AMI, HOM, and ASW scores, UMAP embeddings, and spatial maps compare Garfield with NicheCompass, CellCharter, and GraphST; (f) the mouse olfactory bulb reference shows the meninges, ONL, GL, EPL, MCL, IPL, GCL, and RMS; (g,h) Garfield-derived niches are shown for Stereo-seq and Slide-seqV2 mouse olfactory bulb sections; (i,j) CHAOS scores compare niche resolutions, with the selected resolution indicated in each panel; (k) a Slide-seqV2 query is mapped to a Stereo-seq reference through fine-tuning and niche-label transfer; and (l,m) estimated cell-type proportions across niches and their spatial distributions are shown for representative cell types.

Since this dataset lacks ground truth labels, we qualitatively compared manually identified niches with an anatomical map from the Allen Mouse Brain Reference Atlas [47] (Fig. 3a). These niches showed strong alignment with anatomical subcomponents, accurately reflecting local tissue structures (Fig. 3b). Manually annotated niches displayed unique spatial distributions (Fig. S6a) and were associated with known markers (Fig. S6b), validating their use as pseudo-ground truth for benchmarking. Additionally, niches with similar anatomical structures exhibited comparable variations in cell-type abundance (Fig. S6c), suggesting that differences in cell-type composition, rather than gene expression alone, contribute to biological variation within niches.

Using ARI, NMI, AMI, HOM, and ASW as evaluation metrics, we benchmarked Garfield against competing tools. Garfield consistently outperformed all methods across these metrics (Fig. 3c–e). While all methods could distinguish major structures like CA1, CA2, CA3, and DG, GraphST struggled to resolve finer niche structures such as LP and Stratum. NicheCompass had difficulty identifying the Corpus callosum and exhibited potential over-segmentation, particularly in the LP niche. Both CellCharter and Garfield suggested LP might be homogeneous, but CellCharter’s performance on Stratum was notably weaker than Garfield’s. Garfield delivered the most robust results, accurately characterizing the spatial distribution of all niches in the mouse hippocampus. These findings demonstrate Garfield’s superior ability to detect tissue niches.

### Mapping shared and unique spatial niches in the mouse olfactory bulb using Stereo-seq and Slide-seqV2

Garfield’s ability to identify niches across different spatial sequencing platforms was demonstrated using two mouse olfactory bulb (MOB) spatial transcriptome slices profiled by Slide-seqV2 [6] and Stereo-seq [7] (Fig. 3f–j). Garfield accurately resolved known tissue structures, including the olfactory nerve layer (ONL), glomerular layer (GL), external plexiform layer (EPL), mitral cell layer (MCL), granule cell layer (GCL), internal plexiform layer (IPL), and rostral migratory stream (RMS), based on DAPI-stained image [48] and Allen Mouse Brain Atlas annotations [47] (Fig. 3f).

Notably, Garfield detected finer niche structures in Slide-seqV2 data, such as subdividing the EPL into EPL_inner and EPL_outer. This difference likely reflects the higher capture resolution of Slide-seqV2, highlighting Garfield’s capacity to discern detailed spatial structures. The identified niches exhibited specific spatial distributions and distinct marker gene expressions (Fig. S7). For example, niche 10 (Slide-seqV2) was enriched with Ptgds [49] (meninge [50]), niche 4, 9 (ONL) with S100a5 [51], niche 6 (IPL) with Slc17a7 [52], and niche 7 (RMS) with Mbp [53]. Quantitative analysis [52] confirmed these patterns, showing better spatial continuity and smoothness of niches identified by Garfield across platforms, irrespective of resolution (Fig. 3i and j).

To address the transferability of niches between spatial omics datasets, Garfield used Stereo-seq data as a reference and Slide-seqV2 data as a query. Garfield effectively transferred niche labels from the reference to the query, maintaining consistency in the latent space (Fig. 3k). This result suggests Garfield’s potential for annotation migration across datasets, revealing conserved niches between sequencing technologies. Such findings pave the way for constructing comprehensive 3D spatial maps.

Additionally, we explored cell-type enrichment in different niches using publicly available MOB scRNA-seq data from GSE121891 [51]. Cell-type annotations mapped to Stereo-seq data revealed unique niche-specific characteristics, including neuronal subtypes across adjacent layers, mesenchymal cell subpopulations within the ONL, and oligodendrocyte precursor cells in the RMS (Fig. 3l and m). These findings further demonstrate Garfield’s utility in uncovering complex spatial and cellular relationships within tissues.

### Uncovering heterogeneity in the NSCLC tumor microenvironment using Garfield

**Figure 4:**
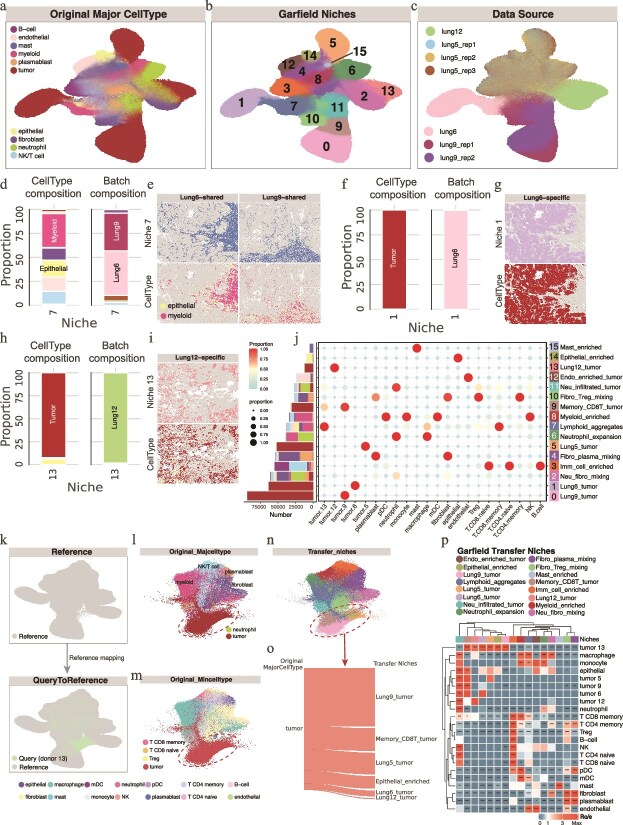
Garfield constructs a spatial atlas of heterogeneous non-small cell lung cancer patients and characterizes differences in cellular abundance and gene signatures among niches: (a–c) UMAPs show major cell types, 16 Garfield-derived niches, and donor or replicate origins; (d,e) cell-type and sample composition together with tissue maps characterize donor-shared niche 7; (f,g) the corresponding analyses characterize Lung 6-specific niche 1; (h,i) the corresponding analyses characterize Lung 12-specific niche 13; (j) a bubble plot summarizes cell-type distribution and abundance across niches, with bubble size representing cell proportion and niche identity indicated by category; (k–n) UMAPs show the reference atlas, mapping of the held-out donor 13 query, its original major and minor cell types, and transferred niche assignments; (o) a Sankey plot traces query tumor cells to transferred niches; and (p) observed-to-expected ratios show preferential cell-type localization across transferred niches, with values greater than 1 indicating enrichment.

To demonstrate Garfield’s ability to resolve tumor microenvironment heterogeneity in large-scale cancer datasets, we applied it to a human non-small cell lung cancer (NSCLC) dataset consisting of eight tissue sections (702 199 cells) from five donors, with two donors having two or three technical replicates, respectively. A spatial atlas of NSCLC was constructed using all samples except donor 13 (625 663 cells). Garfield identified 16 distinct niches driven by differential cell composition (Fig. 4a, Fig. S8b left) and spatial organization (Fig. S8b right). Several tumor-specific niches (e.g. niches 0, 1, 5, 9, and 13) were identified as donor-specific but shared across technical replicates, indicating the removal of batch effects with Garfield (Fig. 4c, Fig. S8b). Notably, niche 7 was found across donors, particularly donors 6 and 9, and defined as a donor-shared niche. It exhibited a distinct spatial distribution in histological images and was enriched in epithelial and myeloid cells (Fig. 4d and e). Additionally, donor-specific niches were observed, such as niche 1 (specific to donor 6, Fig. 4f) and niche 13 (specific to donor 12, Fig. 4h). Spatial location and neighborhood cell compositions corroborated these findings (Fig. 4g, Fig. 4i). Key core genes driving these niches were identified, including EGFR in donor 6-specific niche 1 (Fig. S8c) and S100A6 in donor 12-specific niche 13 (Fig. S8d). Other niches, such as niche 2, were co-enriched with tumor-related fibroblasts and neutrophils, suggesting their relevance to tumor progression (Fig. S8a and b). Analysis of cell-type proportions across niches revealed that each niche could be annotated by a significantly enriched cell type, showcasing Garfield’s ability to highlight tumor heterogeneity in NSCLC (Fig. 4j). These findings were further supported by preference analysis using Ro/e (Fig. S8e).

To evaluate Garfield’s spatial reference mapping capability, we used data from a new donor (donor 13) and mapped them onto the constructed spatial reference using minimal weight-restricted fine-tuning. A k-nearest neighbors classifier trained on Garfield’s latent space transferred niche labels to the query cells, achieving accurate integration while preserving biological features (Fig. 4k–n, Fig. S8f). Label transfer identified a donor 13-specific niche outside the reference, closely related to a tumor-associated niche, with a consistent cell-type distribution (Fig. 4k–o). This indicates Garfield’s ability to detect novel spatial variations while maintaining correlations between reference and query datasets. Taken together, these results highlight Garfield’s capacity to construct large-scale spatial reference atlas and map query spatial datasets to a single-cell resolution spatial atlas with minimal fine-tuning, enabling the discovery of new spatial patterns in complex datasets.

### Garfield identifies heterogeneous niches in human breast cancer 10x Xenium data

**Figure 5:**
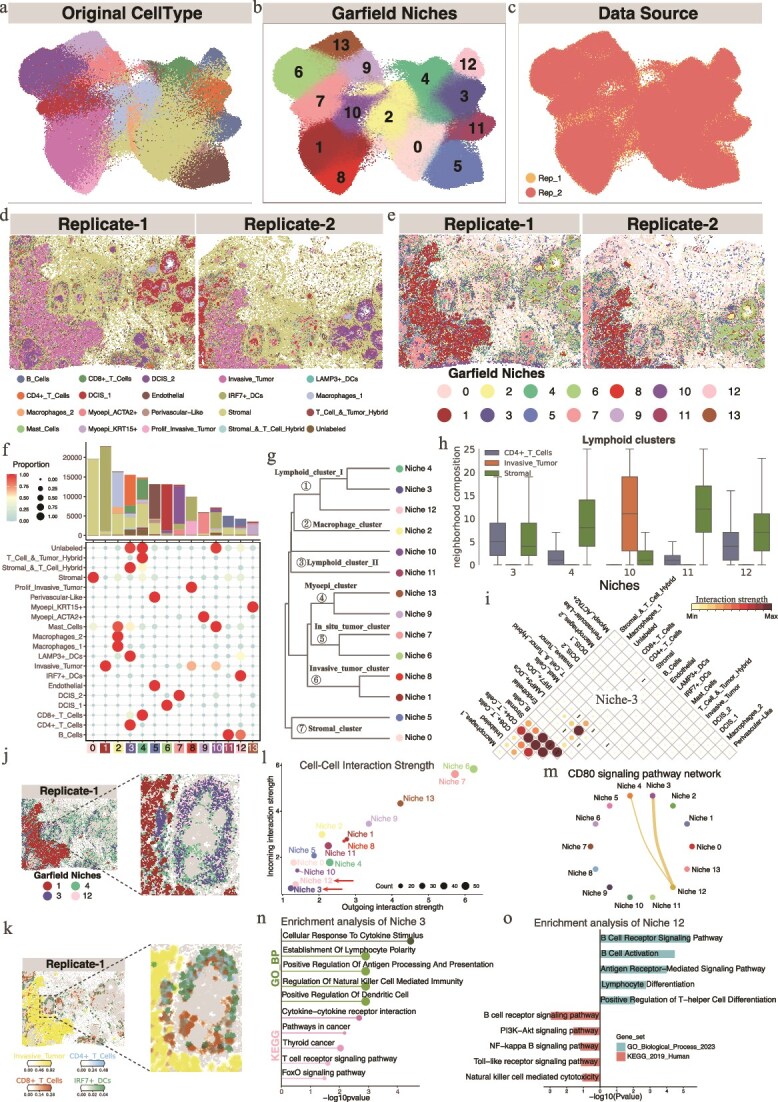
Garfield identifies functionally distinct niches with unique spatial significance in human breast cancer: (a–c) UMAPs of two integrated 313-probe 10x Xenium tissue replicates are grouped by original cell type, 14 Garfield-derived niches, and data source; (d,e) spatial maps show the original cell types and Garfield niches in both replicates; (f) a bubble plot summarizes cell-type abundance across niches, with bubble size denoting cell proportion and niche identity indicated by category; (g) a dendrogram groups molecularly similar niches; (h) boxplots show the composition of the 25 nearest neighboring cells in lymphoid-related niches, with centre lines indicating medians, boxes indicating interquartile ranges, whiskers extending to 1.5 times the interquartile range, and only cell types with an average contribution of 5–60% displayed; (i) a heatmap shows proximal cell–cell interactions within niche 3, with significance assessed by a permutation test (*n = 1000*): * *P* ¡.05, ** *P* ¡.01, *** *P* ¡.001; (j,k) spatial maps show tumor-specific and lymphoid-associated niches and the relative densities of invasive tumor cells, CD8-positive T cells, CD4-positive T cells, and IRF7-positive dendritic cells at the tumor boundary; (l) a scatterplot compares incoming and outgoing interaction strengths across niches; (m) a network shows CD80 signaling interactions, with arrow thickness representing interaction strength; and (n,o) enrichment analyses show the molecular pathways and biological processes associated with niches 3 and 12.

Using Garfield, we analyzed a human breast cancer (BC) dataset profiled with 10x Xenium technology to unravel tumor modular organization and interactions between tumor cells and their microenvironment. Garfield effectively captured biological variation and integrated replicates from the same tissue (Fig. 5a–c). By applying Leiden clustering on Garfield’s latent space, we identified 14 distinct niches with clear boundaries, independent of spatial resolution (Fig. S9a). A closer examination of the niches revealed that each was composed of unique combinations of cell types, underscoring their heterogeneity (Fig. 5a and b, d and e, Fig. S9e). For example, niche 1 and niche 8 were mainly enriched with invasive and proliferating tumor cells, respectively, showing similar spatial distributions across replicates (Fig. 5d and e). We also observed immune cells such as T cells, B cells, and dendritic cells (DCs) were prevalent in niches 3, 4, and 12, suggesting a close association with tumor development. These niches were further supported by well-known marker gene enrichment, validating the layered structure (Fig. S9b).

We further explore cell composition within niches and perform hierarchical clustering based on Garfield latent (Fig. 5f and g). Hierarchical clustering grouped niches with similar cell-type compositions and gene features into different compartments, such as lymphoid_cluster_I (niches 3, 4, and 12), lymphoid_cluster_II (niches 10 and 11), Invasive_tumor_cluster (niches 1 and 8), and so on (Fig. 5g). For lymphoid clusters, we performed neighborhood composition analysis, and confirmed consistent cell-type co-localization within lymphoid clusters (Fig. 5i), aligning with previous observations (Fig. 5a and b). To further explore the functional dynamics of lymphoid niches, we constructed proximal interaction networks for niches 3, 4, and 12 (Method). Strong interactions between CD4+ and CD8+ T cells and stromal cells were identified in these three niches (Fig. 5i, Fig. S9c and d), while niche 12 also showed additional crosstalk between B cells and immune/stromal cells, likely due to its high B cell enrichment (Fig. S9d). Given prior evidence that myoepithelial cells can form barriers to inhibit tumor invasion [54], we hypothesize that these lymphoid niches may form a local immune regulatory network to curb tumor progression.

To substantiate this hypothesis, we firstly visualized tumor-specific niche 1 and lymphoid-associated niches 3, 4, and 12 on tissue sections (Fig. 5j). Notably, these lymphoid-related niches demonstrated a consistent enrichment at the periphery of the invasive tumor niche, forming a discernible aggregation network. This spatial pattern was further corroborated through independent replicates (Fig. S9f). At the cell-type level, significant enrichment of immune cell populations, including CD4+, CD8+ T, and DCs, was observed at the same invasive tumor margins, consistent with prior findings (Fig. 5k, Fig. S9g). These results provide preliminary evidence for the co-localization of diverse immune cell types at the tumor boundaries, likely facilitating the formation of a localized immune regulatory network through niches interactions. To further elucidate this phenomenon, we employed CellChat V2 [55], an advanced tool for inferring cell–cell interactions with integrated spatial data. Analysis across all niches revealed that niches 3 and 12 exhibited minimal incoming and outgoing interaction strengths, indicative of the establishment of a localized interaction network within these regions (Fig. 5l). Moreover, the CD80 signaling pathway was identified as a key regulatory axis driving this local network, underscoring its potential role in modulating immune-tumor dynamics (Fig. 5m). To investigate the underlying mechanisms of this local network at a functional level, we conducted enrichment analysis on the differential markers of niches 3 and 12 (Fig. 5n and o). Both niches were found to be enriched in tumor-related pathways, including antigen-mediated signaling pathway and cell-mediated immunity. Additionally, distinct signaling pathways were identified for each niche, highlighting their unique characteristics. For instance, niche 3 was specifically associated with the cellular response to cytokine stimulus (Fig. 5n), while niche 12 was linked to B cell activation and related processes (Fig. 5o). These findings indicate that while niches 3 and 12 share core functional features, contributing to network synergy, they also possess niche-specific attributes. This balance between shared and unique functions is essential for maintaining the functional diversity and adaptability of the network, thereby supporting its scalability and effectiveness in regulating immune–tumor interactions.

## Discussion

Our findings demonstrate that Garfield, a novel and versatile single-cell and spatial embedding algorithm, provides a robust framework for integrating uni- and multi-modal single-cell and spatial omics data. By leveraging VGAE and a self-supervised, multi-task learning approach, Garfield successfully unifies molecular and spatial information, enabling the precise characterization of cellular landscapes and tissue-specific niches. This capability marks a significant advancement over existing methods, such as Seurat V4, MultiVI, and MOFA+, which rely on more limited (graph) structures and often struggle with spatial and cross-modality integration. The benchmarking results highlight Garfield’s scientific implications, as it consistently outperformed state-of-the-art tools across various datasets, including scRNA + scATAC, scRNA + scADT, and spatial multi-omics.

Particularly in spatial niche detection, Garfield excelled in identifying fine-grained anatomical and functional structures, such as the olfactory bulb’s IPL, tumor-specific niches in NSCLC datasets, and proximal cell–cell interaction network. Garfield revealed novel insight on breast tumor niches by identifing immune niches at tumor margins orchestrating localized CD80-mediated interactions between T cells, B cells, and DCs. These niches formed barrier-like structures around invasive tumor regions, with lymphoid niches enriched for cytokine response (e.g. IFN-*γ*) and B cell activation pathways. Spatial interaction analysis revealed conserved CD80 signaling hubs across replicates, suggesting a coordinated immune surveillance mechanism. These findings underscore its utility in uncovering heterogeneity within complex tissue environments and its ability to integrate information across modalities and resolutions, setting the stage for broad applications in biomedical research.

Garfield’s ability to map query datasets to reference atlases without retraining positions it as a platform for collaborative spatial atlas initiatives. Its capability to construct large-scale spatial reference atlases and seamlessly map query datasets enables a deeper understanding of conserved spatial niches across platforms and conditions. By enabling joint analysis of single-cell and spatial data, Garfield opens avenues to explore conserved architectural principles across tissues and diseases. Extensions to temporal multi-omics or 3D spatial mapping could further illuminate dynamic cellular ecosystems. Another natural extension is missing-modality recovery. Garfield is not evaluated here as a dedicated imputation method, and we therefore make no quantitative imputation claim. However, its variational latent space and omics reconstruction decoder provide a principled starting point: in paired multi-omics data, one could mask an entire modality block at input, infer the latent representation from the observed modality and graph structure, and decode the held-out block for comparison with paired ground truth. A rigorous evaluation would require modality-aware masking, calibration under sparse count data, feature-wise correlation and reconstruction-error metrics, and comparison with dedicated translation or generative models for incomplete or perturbed omics [30, 56]. We therefore treat missing-modality recovery as a future extension rather than as an empirical claim of the present benchmark.

Despite these achievements, some limitations remain. While Garfield efficiently integrates multi-modal data and resolves batch effects, its performance in highly sparse datasets and extreme high-dimensional spaces requires further optimization. Under extreme sparsity, both ApproxSVD-guided augmentation and molecular or spatial graph construction may become less reliable. With few nonzero features per observation, the low-rank reconstruction $\hat{A}=U_{q} S_{q} V_{q}^\top$ may over-smooth weak local structure, and k-nearest-neighbor graphs built from sparse profiles may contain noisier neighborhoods. Practical mitigations include feature selection, tuning the ApproxSVD rank *q* (Supplementary Table S6), and assessing graph-construction neighborhoods, but a systematic study of very-sparse regimes remains future work. For computational resources, all experiments were run on a single NVIDIA A800-SXM4-80GB GPU. Runtime and peak-memory usage across dataset scales (from *∼*7000 to *∼*700 000 observations) are reported in Supplementary Table S3; mini-batch neighbor sampling keeps memory bounded and enabled training of the full *∼*700 000-observation NSCLC atlas in Fig. 4 in about 2 h on one GPU. Additionally, the reliance on reference atlases, such as the Allen Brain Atlas, for validating niche detection introduces potential biases when analyzing previously uncharacterized tissues. Future efforts could focus on enhancing Garfield’s ability to function independently of existing references while improving computational efficiency for large-scale datasets.

Looking ahead, advancing the field of spatial and single-cell multi-omics analysis will require addressing key challenges such as improved scalability, universal applicability across technologies, and deeper insights into dynamic cellular processes. In summary, Garfield bridges a long-standing divide between single-cell and spatial omics, offering a unified framework for hypothesis generation and validation. By providing deeper insights into cellular heterogeneity and spatial organization, it holds immense potential for advancing the understanding of complex biological systems and developing next-generation applications in precision medicine and tissue engineering.

## Methods

### Modeling architecture

#### Data loading and preprocessing

The input to the Garfield model can include one or multiple datasets from various omics sources (such as single-cell RNA, ATAC, ADT, or spatial data), where each row corresponds to a cell and each column represents a feature. Following data loading, Garfield processes the dataset by removing non-informative genes, thereby optimizing the training process. Firstly, genes expressed in fewer than three cells and cells expressing fewer than 100 features are excluded by default. Then, raw counts undergo library-size normalization followed by log transformation. In the case of ATAC and ADT data, term frequency-inverse document frequency (TF-IDF) and Centered Log-Ratio (CLR) normalization methods are applied.

Subsequently, the top 3000 highly variable genes (HVGs) are selected by default and used for dimensionality reduction via principal component analysis (PCA). For chromatin peak data, the top 10 000 highly variable peaks (HVPs) are selected by default, with latent semantic indexing (LSI) employed for dimensionality reduction. Notably, in multi-omics datasets, features derived from PCA or LSI are used as node attributes within the encoder model. This approach has demonstrated exceptional performance in representation learning, as highlighted in the previous study [57]. While in the unimodal scenario, the default is to use original expression profiles as the input of the model.

#### General dataset

A general “GraphAnnTorchDataset” class, inspired by NicheCompass [24], is implemented in Garfield to process single-cell or spatial omics data, enabling graph-based learning by extracting node features, adjacency matrices, and batch labels. In this framework, let $\mathcal{D} = \left \{x_{i}, e_{i}, w_{i}, y_{i} \right \}_{i=1}^{N_{\mathrm{obs}}}$ represent the dataset, where $N_{\mathrm{obs}}$ is the total number of observations. Here, $x_{i} \in \mathbb{R}^{N_{feat}}$ denotes the specific omics feature vector for observation *i*, with *N* features $N_{feat}$, and $e_{i,j} \in \mathbb{R}^{2 \times{N_{edge}}}$ represents the connectivity between observation *i* and its neighbor observation *j*, as derived from the adjacency matrix $\mathbf{A}$. The edge weight $w_{i,j}$ indicates the connectivity weight between observation *i* and its neighbor observation *j*, which is also extracted from $\mathbf{A}$. The $y_{i} \in \mathbb{R}$ represents the batch label for observation *i*. The features $x_{i} \in \mathbb{R}^{N_{feat}}$ represents the expression profile of observation *i* obtained from “adata.X“ or adata.obsm["feat"], and can consist of either raw counts or processed features, supporting the integration of multiple data types (e.g. unimodal or multimodal scenarios).

In the unimodal scenario, we define $\mathbf{x}_{i} = \mathbf{x}_{i}^{(\mathrm{mod}_{j})} \in \mathbb{R}^{N_{\mathrm{mod}_{j}}}$, where $N_{\mathrm{mod}_{j}}$ is the number of features from the specific *j* modality, and *j* refers to one of three common modalities (RNA, ATAC, or ADT). In the multimodal scenario, $\mathbf{x}_{i}$ is a concatenated vector of processed gene expression and chromatin accessibility or protein features. For chromatin peaks, this is represented as $\mathbf{x}_{i} = \mathbf{x}_{i}^{rna} \Vert \mathbf{x}_{i}^{atac}$ with $\mathbf{x}_{i}^{rna} \in \mathcal{R}^{N}_{rna}$, and $\mathbf{x}_{i}^{atac} \in \mathcal{R}^{N}_{atac}$, where $N_{rna}$ and $N_{atac}$ is the number of genes and peak regions, respectively. For protein features, it is expressed as $\mathbf{x}_{i} = \mathbf{x}_{i}^{rna} \Vert \mathbf{x}_{i}^{adt}$ with $\mathbf{x}_{i}^{adt} \in \mathcal{R}^{N}_{adt}$, where $N_{adt}$ is the number of antibody. The adjacency matrix $\mathbf{A} \in \mathbb{R}^{N_{obs} \times N_{obs}}$ is derived from “adata.obsp[“connectivities”].”

#### Construction of neighbor graph

Consider a neighborhood graph $\mathcal{G} = \{v_{i}, s_{i}, x_{i}, \gamma _{i}\}$, where each node $v_{i}$ represents an observation, with the adjacency matrix $s_{i}$ encoding its connectivity to single-cell or spatial neighbors. From this matrix, we can derive the edge relationships and their associated weights between $v_{i}$ and other observations. The vector $x_{i}$ denotes the expression vector of node $v_{i}$, while $\gamma _{i}$ represents its label vector. In the Garfield algorithm, $\mathcal{G}$ is an un-directed neighborhood graph, composed of sample-specific symmetric k-nearest neighbor sub-graphs $\mathcal{G}_{k}$, where *k* is the number of samples. The construction of $\mathcal{G}$ depends on the data type.

For single-cell unimodal data, $\mathcal{G}$ is computed using the “sc.pp. neighbors” function from the Scanpy [33] package. In the single-cell multimodal scenario, $\mathcal{G}$ consists of intra-modality connectivity (intra_connect) and inter-modality connectivity (inter_connect). The calculation of intra_connect is similar to that of the single-cell unimodal case. Inspired by previous studies [58], the connectivity between different modalities are modeled as a linear assignment optimization problem, and inter_connect is obtained by solving this objective function. We extended the original cell-matching method [58, 59] to support not only scRNA-seq and single-cell proteomics but also scRNA-seq and scATAC-seq data. Our approach involves scoring the gene activity in scATAC-seq data prior to initial matching via linear assignment, ensuring that peak features overlap with those of scRNA-seq data. This step is automatically performed by Garfield’s built-in ”gene_scores” function, which was modified by SIMBA [60]. Thus, the final connectivity are computed as the weighted sum of the intra_connect ($\mathcal{E}_{intra}$) and inter_connect ($\mathcal{E}_{inter}$). Mathematically, this can be expressed as


(1) \begin{eqnarray*}& \mathbf{A_{\mathrm{sc}}} = w \cdot \mathcal{E}_{inter} + (1-w) \cdot \mathcal{E}_{intra},\end{eqnarray*}

where the weight *w* represents the relative contributions of $\mathcal{E}_{inter}$ and $\mathcal{E}_{intra}$ to the overall connectivity.

For spatial single- or multi-modal data, Garfield provides four methods to compute spatial connectivity ($\mathcal{E}_{\mathrm{spatial}}$): mu_std, radius, KNN, and Squidpy.

1. mu_std: refer to previous study [23], Garfield computes a full pairwise Euclidean distance matrix between cells based on their spatial coordinates. For each cell, the k-nearest neighbors are identified, and their corresponding distances are analyzed. The boundary for establishing a connectivity is defined as $\mathrm{boundary} = \mu + \sigma$, where *μ* represents the mean and *σ* the standard deviation of the distances to the nearest neighbors. If the distance between two cells is within this boundary, an edge is assigned a weight of 1; otherwise, the weight is 0. This is formalized as
  (2) \begin{eqnarray*} A_{ij} = \begin{cases} 1, & \mathrm{if}\ Dist_{ij} \leq \mathrm{boundary} \\ 0, & \mathrm{otherwise} \end{cases}\end{eqnarray*}
2. Radius: in the Radius method, a radius-based neighbor search is employed. A “NearestNeighbors” object is instantiated with a specified radius parameter, and the function identifies all neighbors for each cell within this radius. The resulting cell pairs are connected in the graph, with edge weights corresponding to their spatial distances.
3. KNN: in the KNN method, the k-nearest neighbors of each cell are identified using the “kneighbors” function, which returns both the distances and the indices of the nearest neighbors. Graph edges are then established between each cell and its k-nearest neighbors, with edge weights representing the spatial distances.
4. Squidpy [34]: Garfield offers an additional method implemented through Squidpy’s “spatial_neighbors” function, which computes spatial connectivity based on physical proximity.

Additionally, based on the spatial single-modal expression profile, we also compute molecular connectivity ($\mathcal{E}_{\mathrm{molecular}}$) using the “sc.pp.neighbors” function similar to single-cell data. This function builds a neighbor graph based on expression data, where each node represents a cell, and edges are determined by similarity in molecular profiles. For spatial multi-modal data, we build upon the aforementioned workflow by adapting established single-cell multi-omics processing methods. Utilizing our optimized cell matching algorithm, we identify cross-modality connectivity within spatial multi-modal datasets. These resulting molecular connectivity graph captures both intra-modality ($\mathcal{E}_{intra}$) and inter-modality ($\mathcal{E}_{inter}$) relationships (if spatial multi-modal data exists), providing a detailed view of interactions at the molecular and spatial levels. As a result, the overall connectivity is computed as the sum of the weights from both spatial connectivity and molecular connectivity. Mathematically, the adjacency matrix $\mathbf{A}_{sp}$ for spatial uni-modal data is defined as


(3) \begin{eqnarray*}& \mathbf{A}_{sp} = (1 - w) \cdot \mathcal{E}_{intra} + w \cdot \mathcal{E}_{spatial}\end{eqnarray*}

For spatial multi-modal data, it is expressed as


(4) \begin{eqnarray*}& \mathbf{A}_{sp} = (1 - w) \cdot \mathcal{E}_{molecular} + w \cdot \mathcal{E}_{spatial},\end{eqnarray*}

where $\mathcal{E}_{molecular}$ is defined by


(5) \begin{eqnarray*}& \mathcal{E}_{molecular} = \mathcal{E}_{intra} + \mathcal{E}_{inter}\end{eqnarray*}

### Garfield model

#### Model overview

To address the scarcity and task-specific nature of label information, we propose learning embeddings in an unsupervised manner to enhance model generalizability. Garfield begins by encoding all observations into a unified graph, where nodes represent cells or spatial units and edges capture molecular or spatial relationships. It then applies a powerful representation learning approach, the VGAE [31], incorporating a SVD-based graph contrastive strategy, an optional MMD-based transformation, and a multi-task learning setup. This setup includes both node- and edge-level tasks, embedding observations into a low-dimensional, batch-invariant biological space ideal for batch effect correction and multi-omics integration at the single-cell and spatial levels.

The model is built on an encoder–decoder framework. Specifically, the encoder consists of a graph encoder module, while the decoder includes a graph decoder module to reconstruct the adjacency matrix from the latent vector, and a feature reconstruction decoder module for handling omics modalities. This dual-module structure ensures that the latent representations capture both physical graph and molecular information while integrating spatial data. Throughout, Garfield is trained in a fully self-supervised manner: cell-type and niche labels are never used during training and serve only for *post hoc* evaluation of the unsupervised embedding. The only internal data split is on graph edges (via the edge_val_ratio/node_val_ratio parameters), used solely for early stopping of the VGAE, so node features and labels are neither split nor leaked. In the query-to-reference experiments, the query dataset (e.g. the NSCLC donor lung13) is held out entirely from atlas construction and mapped only afterwards, with labels transferred *post hoc* by a kNN classifier in the learned latent space.

Inspired by the LightGCL [39] framework, Garfield uses a GAT backbone to automatically extract local graph dependencies. The SVD-guided augmentation enhances graph contrastive learning by analyzing global collaborative relations, improving single-cell representation. Additionally, an optional drop edge augmentation strategy is available.

In line with variational autoencoder standards, we use a standard normal distribution as the prior for latent random variables, denoted as $Z\sim \mathcal{N}(0,1)$, and apply the reparameterization trick, enabling end-to-end training through backpropagation.

#### Data augmentation

Contrastive learning aims to capture invariant representations between similar and dissimilar data pairs. To create similar pairs, we apply data augmentation to generate perturbed views of the input data. We offer two graph augmentation methods: ApproxSVD-guided augmentation [39] (default) and edge-drop augmentation [61].

For ApproxSVD-guided augmentation, we perform a sparse ApproxSVD on the original adjacency matrix *A* as $A = U S V^\top$. Here, *U* and *V* are orthonormal matrices representing the eigenvectors of the row–row and column–column correlation matrices of *A*, while *S* is a diagonal matrix of singular values. The largest singular values typically correspond to the principal components of the graph. We truncate the singular values, keeping only the top *q*, and reconstruct the adjacency matrix as $\hat{A} = U_{q} S_{q} V_{q}^\top$, where $U_{q} \in R^{m \times q}$ and $V_{q} \in R^{n \times q}$ contain the first *q* columns of *U* and *V*, respectively, and $S_{q} \in R^{q \times q}$ is the diagonal matrix of the top *q* singular values. The reconstructed matrix $\hat{A}$ is a low-rank approximation of *A*, where $\mathrm{rank}(\hat{A}) = q$. The benefits of SVD-based augmentation are two-fold: it emphasizes principal components, highlighting critical cell–cell interactions, while preserving global collaborative signals across cell pairs. To handle large adjacency matrices efficiently, we use the randomized SVD algorithm from Halko *et al*. [62], which approximates the input matrix using a low-rank orthonormal matrix and then applies SVD on the smaller matrix:


(6) \begin{eqnarray*}& \hat{U}_{q}, \hat{S}_{q}, \hat{V}_{q}^\top = \mathrm{ApproxSVD}(A, q), \quad \hat{A}_{\mathrm{SVD}} = \hat{U}_{q} \hat{S}_{q} \hat{V}_{q}^\top ,\end{eqnarray*}

where *q* is the desired rank, and $\hat{U}_{q} \in \mathbb{R}^{m \times q}$, $\hat{S}_{q} \in \mathbb{R}^{q \times q}$, and $\hat{V}_{q} \in \mathbb{R}^{n \times q}$ are the approximated matrices.

For edge-drop augmentation (optional), we randomly remove edges from $\mathcal{E}$ based on a dropout ratio $p_{\mathrm{drop}}$. Specifically, we sample an indicator matrix $R_{\mathrm{edge}{\_}\mathrm{mask}} \in \mathbb{R}^{N \times N}$ to determine which edges to mask. Each entry $R_{ij}$ is sampled as


(7) \begin{eqnarray*}& R_{ij} \sim \mathrm{Bernoulli}(1 - p_{\mathrm{drop}}) \text{ if}\ A_{ij} = 1, \text{ else}\ R_{ij} = 0\end{eqnarray*}

The augmented adjacency matrix is then computed as


(8) \begin{eqnarray*}& \hat{A} = A \odot R_{\mathrm{edge}{\_}\mathrm{mask}} ,\end{eqnarray*}

where *⊙* denotes the Hadamard product.

Both ApproxSVD-guided and edge-drop augmentations generate perturbed adjacency matrices $\hat{A}$ that retain the structure of the original graph. To further enhance robustness, we introduce a feature masking vector $R_{\mathrm{feat}{\_}\mathrm{mask}} \in \mathbb{R}^{N \times 1}$ (denoted as $R_{\mathrm{fm}}$), where each entry $R_{i} \sim \mathrm{Bernoulli}(1 - p_{\mathrm{drop}})$, with $p_{\mathrm{drop}}$ as the masking probability. The masked feature matrix is expressed as


(9) \begin{eqnarray*}& \hat{X} = \mathrm{Concat}(x_{1} \odot R_{\mathrm{fm}}, x_{2} \odot R_{\mathrm{fm}}, \dots, x_{N} \odot R_{\mathrm{fm}})\end{eqnarray*}

#### Garfield encoder

Through data processing and ApproxSVD-guided or edge-drop augmentation strategies, Garfield could generate an original view $\mathcal{G}_{1}$ as well as augmented view $\mathcal{G}_{2}$; we then learn cell embeddings using node-level graph contrastive learning. Given that GAT [37] is a state-of-the-art architecture for graph representation learning and excels at extracting rich embeddings from graph-structured data, we employ it as the feature extractor to capture the latent embedding of the cell nodes. Specifically, in GAT, each node updates its representation by attending to its neighbors, using its own representation as the query. This approach extends the standard neighbor averaging or max-pooling by allowing nodes to compute a weighted average, prioritizing relevant neighbors [63].

For a set of node features $h = [h_{1}; h_{2}; \dots ; h_{N}]$, where $h_{i} \in \mathbb{R}^{D}$, a shared linear transformation with a weight matrix *W* is applied to all nodes. An attention mechanism *α* is then applied pairwise to the transformed node features via $e_{ij} = \alpha (W h_{i}, W h_{j})$, which reflects how much node *j* contributes to node *i*. The attention coefficients for head *n*, $\alpha _{ij}^{(n)}$, are computed as


(10) \begin{eqnarray*}& \alpha_{ij}^{(n)} = \frac{\exp(\mathrm{LeakyReLU}(\mathbf{a}^{(n)^{T}} [W h_{i} \| W h_{j}]))}{\sum_{k \in \mathcal{N}_{i}} \exp(\mathrm{LeakyReLU}(\mathbf{a}^{(n)^{T}} [W h_{i} \| W h_{k}]))},\end{eqnarray*}

where ∥ denotes concatenation and LeakyReLU is used to allow a small gradient when the argument is non-positive. The normalized coefficients are then combined with the neighboring features (often after applying a non-linearity *σ*) to produce the output node features.

Multi-head attention, denoted as *K*, can be applied in two ways:


(11) \begin{eqnarray*}& \tilde{h}_{i} = \bigg\|_{k=1}^{K} \sigma \left( \sum_{j \in \mathcal{N}_{i}} \alpha_{ij}^{k} W^{k} h_{j} \right)\!, \quad \hat{h}_{i} = \sigma \left( \frac{1}{K} \sum_{k=1}^{K} \sum_{j \in \mathcal{N}_{i}} \alpha_{ij}^{k} W^{k} h_{j} \right)\end{eqnarray*}

The left formulation is suitable for hidden layers, as it outputs concatenated representations of dimension *K · D*, while the right formulation, which averages the attention heads, is appropriate for the output layer.

In addition to GAT, we also provide GATv2 [36], an improved version with a dynamic attention mechanism. The attention coefficients in GATv2 are calculated as


(12) \begin{eqnarray*}& \alpha_{ij}^{(n)} = \frac{\exp \left( a^{(n)^{T}} \mathrm{LeakyReLU} \left( W h_{i} \| W h_{j} \right) \right)}{\sum_{k \in \mathcal{N}(i)} \exp \left( a^{(n)^{T}} \mathrm{LeakyReLU} \left( W h_{i} \| W h_{k} \right) \right)}\end{eqnarray*}

For each node *i*, the updated means in layer *l* are given by


(13) \begin{eqnarray*}& \mu_{i}^{(l)} = \frac{1}{N_{\mathrm{head}}} \sum_{n=1}^{N_{\mathrm{head}}} \left[ \sum_{j \in \mathcal{N}(i)} \alpha_{ij}^{(n)} W h_{j}^{(l-1)} \right],\end{eqnarray*}

where $\mu _{i}^{(l)}$ and $\mu _{i}^{(l-1)}$ represent the means in the *l*th and *(l-1)*th layers, respectively, and $\mathcal{N}(i)$ refers to the neighbors of node *i*. Stacking *L* graph layers results in *L* outputs $[\mu _{1}, \mu _{2}, \dots , \mu _{L}]$, from which we compute the final means and variances as


(14) \begin{eqnarray*}& \mu = \frac{1}{L} \sum_{l=1}^{L} \mu^{l}, \quad \sigma = \mathrm{MLP}(\mu)\end{eqnarray*}

The variances are learned via a one-layer multilayer perceptron (MLP): $\sigma = \exp (\mu \mathbf{W} + \mathbf{b})$, where $\mathbf{W} \in \mathbb{R}^{d \times d}$ and $\mathbf{b} \in \mathbb{R}^{d}$ are learnable parameters. To generate latent representations $z_{i}$, we sample from $\mathcal{N}(\mu _{i}, \sigma _{i}^{2})$ using the reparameterization trick: $z_{i} = \mu _{i} + \sigma _{i} \cdot \varepsilon$ where $\varepsilon \sim \mathcal{N}(0, \mathbf{I})$ is Gaussian noise. Although the standard deviation is computed as a deterministic function of the mean rather than emitted by an independent encoder head, the approximate posterior $q(z\mid x)=\mathcal{N}\!\left (\mu (x),\,\mathrm{diag}(\sigma (x)^{2})\right )$ remains a diagonal Gaussian for every input because *σ* is an element-wise exponential of a vector. The closed-form Kullback–Leibler (KL) divergence to the $\mathcal{N}(0,\mathbf{I})$ prior, $\mathrm{KL}=\tfrac{1}{2}\sum _{d}\!\left (\sigma _{d}^{2}+\mu _{d}^{2}-1-2\log \sigma _{d}\right )$ (Equation 33), depends on the posterior being diagonal Gaussian, not on *μ* and *σ* being parameterized independently. Thus, the closed-form KL, the reparameterization trick, and the corresponding Evidence Lower Bound (ELBO) term remain valid under this formulation. Tying *σ* to *μ* yields a per-node adaptive variance that scales with the encoded signal. The separate log-standard-deviation head present in the canonical VGAE encoder template is not used by Garfield’s training forward pass or by the reported inference embeddings; we clarify this implementation detail and deprecate the unused head in the revised code to avoid ambiguity.

For Garfield Light, a low-memory version of the model, we replace the graph attention layers with graph convolutional layers [38] for message passing. Additionally, to reduce the dimensionality of $x_{i}$, an optional fully connected layer with hidden size $N_{\mathrm{hid}}$ can be added before the graph encoder network to lower memory consumption. Its node-wise formulation is


(15) \begin{eqnarray*}& h_{i} = \mathrm{ReLU}(\mathbf{W} \mathbf{x}_{i}) ,\end{eqnarray*}

where $\mathbf{W} \in \mathbb{R}^{N_{\mathrm{hid}} \times N_{\mathrm{feat}}}$ is a learnable weight matrix (bias terms are omitted for simplicity).

#### Garfield decoder

After estimating the probability distribution of the latent variables *Z*, the goal of graph generation is to reconstruct both the original graph structure and expression profiles. To achieve this, the Garfield decoder consists of a graph decoder module and an omics decoder module. The omics decoder module is further divided into two sub-modules: the inner product decoder and the feature reconstruction decoder.

For the graph decoder module, the reconstructed adjacency matrix $\tilde{A}$ is computed using the cosine similarity between pairwise latent features, formulated as


(16) \begin{eqnarray*}& \tilde{A}_{i,j} = \mathrm{cosine}\ \mathrm{sim}(z_{i}, z_{j}) = \frac{z_{i} \cdot z_{j}}{|z_{i}||z_{j}|}\end{eqnarray*}

In the omics decoder module, we firstly use the inner product decoder to reconstruct the graph’s adjacency matrix. Given the node embeddings $Z \in \mathbb{R}^{N \times d}$, where *N* is the number of nodes and *d* is the embedding dimension, the inner product decoder predicts edges by calculating the dot product between node embeddings. The predicted adjacency matrix $\tilde{A^{\prime}}$ is expressed as


(17) \begin{eqnarray*}& \tilde{A^{\prime}} = \sigma(ZZ^{T}),\end{eqnarray*}

where $Z \in \mathbb{R}^{N \times d}$ is the embedding matrix, with each row representing a node’s embedding. The product $ZZ^{T}$ results in an *N × N* matrix, where each element $\tilde{A^{\prime}}_{i,j}$ represents the predicted probability of an edge between nodes *i* and *j*. The activation function *σ* (commonly sigmoid) constrains output values to the range [0, 1], making them interpretable as edge probabilities.

The second sub-module, the feature reconstruction decoder, aims to reconstruct the omics expression matrix $\tilde{X}$ from the embeddings *Z*. In the present experiments, this decoder reconstructs the observed feature matrix used for training. Garfield is therefore not trained or evaluated as a missing-modality imputation model in this study. Recovering a fully masked modality would require modality-specific masking or output heads and a separate validation protocol against paired ground truth. Given two nodes *i* and *j* with embeddings $h_{i}$ and $h_{j}$, the attention coefficient between them is computed as


(18) \begin{eqnarray*}& e_{ij} = \mathrm{LeakyReLU}\left(a^\top\left[W h_{i} \| W h_{j}\right]\right)\!,\end{eqnarray*}

where *a* is a learnable attention vector, *W* is the weight matrix, and ∥ denotes concatenation. The normalized attention coefficients are then calculated using the softmax function:


(19) \begin{eqnarray*}& \alpha_{ij} = \frac{\exp(e_{ij})}{\sum_{k \in \mathcal{N}(i)} \exp(e_{ik})}\end{eqnarray*}

The node embedding is updated as a weighted sum of its neighbors’ embeddings:


(20) \begin{eqnarray*}& h_{i}^{\prime} = \sigma\left(\sum_{j \in \mathcal{N}(i)} \alpha_{ij} W h_{j}\right)\!,\end{eqnarray*}

where *σ* is an activation function (e.g. ReLU), and $\mathcal{N}(i)$ is the set of neighboring nodes of *i*. If edge weights *w* are used, they scale the node features during message passing:


(21) \begin{eqnarray*}& h_{i}^{\prime} = \sigma\left(\sum_{j \in \mathcal{N}(i)} \alpha_{ij} \cdot w_{ij} W h_{j}\right)\!,\end{eqnarray*}

where $w_{ij}$ is the edge weight between nodes *i* and *j*. By stacking multiple GAT layers and applying attention-based message passing, the decoder reconstructs node features from the latent representations learned during encoding.

#### Garfield data loaders

To scale Garfield to large datasets, where neighborhood graphs $\mathcal{G}$ can become computationally intensive, we use mini-batch training with inductive neighbor sampling [63]. Unlike methods that load the entire graph into memory [20, 64, 65], which is infeasible for large datasets, we sample a fixed number of neighbors $n_{\mathrm{sam}} = 3$ from the *k*-nearest neighbors in $\mathcal{G}$ for message passing during training. Here, $n_{\mathrm{sam}}$ controls the number of neighbors sampled for mini-batch message passing and is distinct from the graph-construction neighbor count *k*. A single-seed sensitivity analysis over $n_{\mathrm{sam}} \in \{1,3,5,10,15\}$ (Supplementary Table S5) shows stable performance around the default value in the tested datasets, whereas larger values increase memory and runtime without a consistent gain.

Similar to NicheCompass [24], we employ two separate task-specific data loaders: a node-level loader for node-level tasks and an edge-level loader for edge-level tasks. Each model iteration includes a forward pass through each loader and a joint backward pass for simultaneous gradient computation. The node-level loader is responsible for omics prediction tasks, such as reconstructing $\tilde{X}$ and the adjacency matrix $\tilde{A}$. The edge-level loader is used solely for reconstructing $\tilde{A}$.

#### Loss function

The Garfield loss function is composed of edge-level and node-level losses because of edge-level and node-level tasks. The edge-level loss utilizes a weighted binary cross-entropy (BCE) to reconstruct edges in the adjacency matrix $\tilde{A}$, while the node-level loss consists of several components: the mean squared error (MSE) for feature reconstruction $\tilde{X}$, contrastive loss for latent vectors, the KL divergence between the variational posteriors and standard normal priors of latent variables, and the batch effect alignment loss for omics data (when multiple batches are present). Furthermore, to ensure the model captures edge-related information in the node-level tasks, a BCE loss with negative sampling is also applied. Among these, the graph reconstruction loss (BCE) and KL divergence loss are jointly referred to as the ELBO.

For edge-level loss, Garfield firstly calculates edge reconstruction using a weighted BCE approach to ensure numerical stability. The positive weight $\mathcal{W}_{\mathrm{pos}}$ is determined by the ratio of negative to positive edges:


(22) \begin{eqnarray*} \mathcal{W}_{\mathrm{pos}} = \frac{\mathcal{E}_{\mathrm{neg}}}{\mathcal{E}_{\mathrm{pos}}} = \frac{\sum (\mathcal{E}_{\mathrm{recon}} == 0)}{\sum (\mathcal{E}_{\mathrm{recon}} == 1)}\end{eqnarray*}

This weighting prevents bias toward negative samples. The mini-batch BCE loss for edge reconstruction is given by


(23) \begin{eqnarray*} \mathcal{L}_{(\mathrm{edge})} =& -\frac{1}{|\mathcal{E}_{\mathrm{recon}}|} \sum_{(i,j) \in \mathcal{E}_{\mathrm{recon}}} \left[ \omega_{\mathrm{pos}} A_{i,j} \log(\sigma(\tilde{A}_{i,j})) \right. \nonumber \\ &\left. +\, (1 - A_{i,j}) \log(1 - \sigma(\tilde{A}_{i,j})) \right],\end{eqnarray*}

where $\tilde{A}$ is the edge reconstruction logits matrix, and $\sigma (x) = \frac{1}{1 + e^{-x}}$ is the sigmoid function applied to logits.

Garfield learns the reconstructed expression profile using a self-supervised loss that combines the MSE from the latent embedding (*Z*) with the omics loss function:


(24) \begin{eqnarray*}& \mathcal{L}_{(\mathrm{omics})} = \frac{1}{N} \sum_{i=1}^{N} \| \boldsymbol{x}_{i} - \tilde{\boldsymbol{x}}_{i} \|_{2} ,\end{eqnarray*}

where $\boldsymbol{x}_{i}$ and $\tilde{\boldsymbol{x}}_{i}$ represent the original and reconstructed expression profiles, respectively, *N* is the total number of cells, and $\| \cdot \|_{2}$ denotes the L2 norm (Euclidean distance), which measures the magnitude of the difference between the original and reconstructed profiles.

For contrastive learning [66, 67] of node-level task, latent embeddings $Z_{1}$ (from the original view) and $Z_{2}$ (from the augmented view) are projected into a common latent space using a weight-sharing projection head $g_{I}(\cdot )$, implemented as a two-layer MLP. Given a mini-batch of size *N*, Garfield generates 2N samples $\{x_{1}^{m},x_{2}^{m},\ldots ,x_{N}^{m},x_{1}^{n},x_{2}^{n},\ldots ,x_{N}^{n}\}$ through augmentations (such as SVD-based or edge-drop strategies), forming pairs of original and augmented samples $\{x_{i}^{m}, x_{i}^{n}\}$. The similarity between pairs is computed using cosine distance:


(25) \begin{eqnarray*}& sim(z_{i}^{k_{1}}, z_{j}^{k_{2}}) = \frac{\big(z_{i}^{k_{1}}\big)\big(z_{j}^{k_{2}}\big)^\top}{\big\|z_{i}^{k_{1}}\big\|\big\|z_{j}^{k_{2}}\big\|}, \quad k_{1}, k_{2} \in \{m, n\}, \quad i, j \in [1, N]\end{eqnarray*}

The contrastive loss for a sample $x_{i}^{m}$ is given by


(26) \begin{eqnarray*} \ell_{i}^{m} &= - \log \frac{\exp(sim(z_{i}^{m}, z_{i}^{n}) / \tau_{ins})}{D_{i}^{m}}, \nonumber \\ D_{i}^{m} &= \exp(sim(z_{i}^{m}, z_{i}^{n}) / \tau_{ins}) + \sum_{j \ne i}^{N} \exp(sim(z_{i}^{m}, z_{j}^{m}) / \tau_{ins}) \notag\nonumber \\ &\quad + \sum_{j \ne i}^{N} \exp(sim(z_{i}^{m}, z_{j}^{n}) / \tau_{ins}), \end{eqnarray*}

where $\tau _{ins}$ is the temperature parameter for instance-level similarity. The overall instance-level contrastive loss [68] is computed as


(27) \begin{eqnarray*}& \mathcal{L}_{(\mathrm{ins})} = \frac{1}{2N} \sum_{i=1}^{N} \left( \ell_{i}^{m} + \ell_{i}^{n} \right)\end{eqnarray*}

By minimizing this loss, positive pairs are drawn closer while negative pairs are pushed apart, preserving biological variability and implicitly correcting batch effects [65]. The instance-level objective in Equation 27 is an InfoNCE estimator [68]. Each term $\ell _{i}^{m}$ is the cross-entropy of identifying the matching augmented view among the *2N-1* in-batch candidates; therefore, $\mathcal{L}_{(\mathrm{ins})}=-I_{\mathrm{NCE}}(z_{1};z_{2})+\log (2N-1)$, and minimizing it maximizes the variational lower bound $I_{\mathrm{NCE}}(z_{1};z_{2})\le I(z_{1};z_{2})$ between the original and ApproxSVD-denoised views. This bound is a variational proxy rather than an unbiased MI estimate. Empirically (Supplementary Fig. S10), optimizing the objective raises the bound in the projection space on which the contrastive loss acts, and a shuffled-positive-pair control collapses it. When measured on encoder latents, however, the two views are already strongly mutually informative even without the contrastive term, indicating that on these data the contrastive loss regularizes, rather than solely creates, view-consistent representations.

In addition to instance-level loss, Garfield also employs cluster-level contrastive loss [69], which has shown robust performance in representation learning. Using a two-layer MLP $g_{C}(\cdot )$, the feature matrix is projected into an *M*-dimensional space (where *M* is the number of clusters). The similarity between cluster pairs is also measured using cosine distance:


(28) \begin{eqnarray*}& sim\big(\hat{y}_{i}^{k_{1}}, \hat{y}_{j}^{k_{2}}\big) = \frac{\big(\hat{y}_{i}^{k_{1}}\big)^\top \big(\hat{y}_{j}^{k_{2}}\big)}{\big\|\hat{y}_{i}^{k_{1}}\big\| \big\|\hat{y}_{j}^{k_{2}}\big\|}, \quad k_{1}, k_{2} \in \{a, b\}, \quad i, j \in [1, M]\end{eqnarray*}

The cluster-level loss for a given cluster $\hat{y}_{i}^{a}$ is formulated as


(29) \begin{eqnarray*}& \hat{\ell}_{i}^{a} = - \log \frac{\exp(sim\big(\hat{y}_{i}^{a}, \hat{y}_{i}^{b}) / \tau_{clu}\big)}{\sum_{j=1}^{M} \left[ \exp(sim\big(\hat{y}_{i}^{a}, \hat{y}_{j}^{a}\big) / \tau_{clu}) + \exp(sim\big(\hat{y}_{i}^{a}, \hat{y}_{j}^{b}\big) / \tau_{clu}) \right]},\end{eqnarray*}

where $\tau _{clu}$ is the temperature parameter for cluster-level similarity. The overall cluster-level contrastive loss is


(30) \begin{eqnarray*}& \mathcal{L}_{(\mathrm{clu})} = \frac{1}{2M} \sum_{i=1}^{M} \left( \hat{\ell}_{i}^{a} + \hat{\ell}_{i}^{b} \right) - H(Y),\end{eqnarray*}

with entropy *H(Y)* of cluster assignment probabilities defined as


(31) \begin{eqnarray*}& H(Y) = - \sum_{i=1}^{M} \left[ P\big(\hat{y}_{i}^{a}\big) \log P\big(\hat{y}_{i}^{a}\big) + P\big(\hat{y}_{i}^{b}\big) \log P\big(\hat{y}_{i}^{b}\big) \right]\end{eqnarray*}

and the probability $P(\hat{y}_{i}^{k})$ for a mini-batch under each augmentation calculated by


(32) \begin{eqnarray*}& P(\hat{y}_{i}^{k}) = \frac{\sum_{t=1}^{N} Y_{ti}^{k}}{\|Y^{k}\|_{1}}, \quad k \in \{a, b\},\end{eqnarray*}

where $Y^{a} \in \mathbb{R}^{N \times M}$ represent the output of $g_{C}(\cdot )$ for a mini-batch under the original view, with $Y^{b}$ denoting the corresponding output under the augmented view. This term *H(Y)* helps avoid trivial solutions, ensuring a balanced cluster assignment [70].

The mini-batch KL divergence [24] is composed of two parts: the node-level KL divergence, which involves nodes from the node batch, and the edge-level KL divergence, which includes nodes from both positive and negative edge pairs in the edge batch:


(33) \begin{eqnarray*} \mathcal{L}_{(\mathrm{KL})} = &\frac{1}{N_{\mathcal{V}_{\mathrm{bat}}}} \sum_{i \in \mathcal{V}_{\mathrm{bat}}} \mathcal{L}^{(\mathrm{KL})}_{i}(\mu_{i}, \sigma_{i}; \mathbf{x}_{i}) \notag \\ &+ \frac{1}{4N_{\mathcal{E}_{\mathrm{bat}}}} \sum_{(i,j) \in \mathcal{E}_{\mathrm{bat}}} \left( \mathcal{L}^{(\mathrm{KL})}_{i}(\mu_{i}, \sigma_{i}; \mathbf{x}_{i}) + \mathcal{L}^{(\mathrm{KL})}_{j}(\mu_{j}, \sigma_{j}; \mathbf{x}_{j}) \right)\end{eqnarray*}

Here, the variational parameters $\mu _{i}$ represents the estimated mean of the approximate posterior normal distribution, obtained as outputs from the graph encoder module, and $\sigma _{i}$ represents the estimated standard deviation through one-layer MLP projection based on $\mu _{i}$. $N_{\mathcal{V}_{\mathrm{bat}}}$ represents the number of nodes in the node batch $\mathcal{V}_{\mathrm{bat}}$, while $N_{\mathcal{E}_{\mathrm{bat}}}$ denotes the number of edges in the edge batch $\mathcal{E}_{\mathrm{bat}}$.

Additionally, a similar edge reconstruction loss $\mathcal{L^{\prime}}_{\mathrm{edge}}$ is applied in the node-level tasks using BCE with negative sampling, where $\hat{y}^{\mathrm{pos}}$ represents the decoder output for positive (real) edges, $\hat{y}^{\mathrm{neg}}$ for negative (randomly sampled) edges, and $\mathcal{E}$ is the total number of edges:


(34) \begin{eqnarray*}& \mathcal{L^{\prime}}_{(\mathrm{edge})} = -\frac{1}{\mathcal{E}} \left( \sum_{i} \log\big(\hat{y}_{i}^{\mathrm{pos}}\big) + \sum_{j} \log\big(1 - \hat{y}_{j}^{\mathrm{neg}}\big) \right)\end{eqnarray*}

If the input data contain multiple batches, Garfield employs the MMD [40] loss to align data across different batches. Inspired by scArches [32], Garfield uses MMD to match latent distributions $q_{\phi }(Z \mid s = i)$ and $q_{\phi }(Z \mid s = j)$, where *s* represents the batch key. The MMD loss is formulated as follows:


(35) \begin{eqnarray*}& \mathcal{L}_{(\mathrm{mmd})} = \ell_{\mathrm{mmd}}(\mathbf{Z}_{S=i}, \mathbf{Z^{\prime}}_{S=j}),\end{eqnarray*}

where


(36) \begin{eqnarray*} \ell_{\mathrm{mmd}}(\mathbf{X}, \mathbf{X}^{\prime}) = &\frac{1}{n_{0}^{2}} \sum_{n=1}^{n_{0}} \sum_{m=1}^{n_{0}} k(x_{n}, x_{m}) + \frac{1}{n_{1}^{2}} \sum_{n=1}^{n_{1}} \sum_{m=1}^{n_{1}} k(x_{n}^{\prime}, x_{m}^{\prime}) \notag \\ &- \frac{2}{n_{0} n_{1}} \sum_{n=1}^{n_{0}} \sum_{m=1}^{n_{1}} k(x_{n}, x_{m}^{\prime})\end{eqnarray*}

Here, $\mathbf{X}$ and $\mathbf{X}^{\prime}$ are high-dimensional observations under conditions $S_{i}$ and $S_{j}$, respectively.

The overall loss function of the Garfield model that is optimized during model training is thus


(37) \begin{align*} \mathcal{L}^{\mathrm{Batch}}_{(\mathrm{total})} = \ &\mathcal{L}_{(\mathrm{KL})} \notag \\ &+\,\lambda_{1}^{(\mathrm{edge})} \mathcal{L}_{(\mathrm{edge})} \notag \\ &+\,\lambda_{2}^{(\mathrm{omics})} \mathcal{L}_{(\mathrm{omics})} \notag \\ &+\,\lambda_{3}^{(\mathrm{edge})} \mathcal{L^{\prime}}_{(\mathrm{edge})} \end{align*}

(37) \begin{eqnarray*}\qquad\quad\ \ \ &+\,\lambda_{4}^{(\mathrm{ins})} \mathcal{L}_{(\mathrm{ins})} \notag \\ &+\,\lambda_{5}^{(\mathrm{clu})} \mathcal{L}_{(\mathrm{clu})} \notag \\ &+\,\lambda_{6}^{(\mathrm{mmd})} \mathcal{L}_{(\mathrm{mmd})} \end{eqnarray*}

If no batch information is present, the total loss is as follows:


(38) \begin{eqnarray*} \mathcal{L}^{\mathrm{NoBatch}}_{(\mathrm{total})} = \ &\mathcal{L}_{(\mathrm{KL})} \notag \\ &+\,\lambda_{1}^{(\mathrm{edge})} \mathcal{L}_{(\mathrm{edge})} \notag \\ &+\, \lambda_{2}^{(\mathrm{omics})} \mathcal{L}_{(\mathrm{omics})} \notag \\ &+\, \lambda_{3}^{(\mathrm{edge})} \mathcal{L^{\prime}}_{(\mathrm{edge})} \notag \\ &+\, \lambda_{4}^{(\mathrm{ins})} \mathcal{L}_{(\mathrm{ins})} \notag \\ &+\,\lambda_{5}^{(\mathrm{clu})} \mathcal{L}_{(\mathrm{clu})},\end{eqnarray*}

where the weighting factors for the total loss components are denoted as *λ*. Loss weights were selected at the task-regime level to balance the effective magnitudes of the corresponding terms, rather than by tissue-specific grid search. These effective magnitudes depend on input dimensionality, graph density, mini-batch node/edge sampling, and fixed implementation-level loss-normalization factors. Accordingly, paired multi-omics, spatial, and multi-batch analyses use regime-appropriate settings, while ablation and sensitivity experiments within each regime changed only the targeted module or weight. Because reconstruction losses are evaluated with mean reduction, the implementation rescales them before applying *λ*: the feature-reconstruction MSE is multiplied by a feature-scale factor to approximate a sum-over-features scale comparable with the KL term, and the latent adjacency- and edge-reconstruction terms are multiplied by fixed graph-scale factors. These deterministic constants are absorbed into the effective *λ*. Module ablations (Supplementary Table S4, two seeds) and sensitivity analyses over individual loss weights (Supplementary Table S7, single seed), $n_{\mathrm{sam}}$ (Supplementary Table 5, single seed), ApproxSVD rank *q* (Supplementary Table S6, single seed), and MMD batch alignment (Supplementary Table S8, single seed) support local robustness around the chosen task-level settings.

### Benchmarking

All benchmarking experiments are fully reproducible and can be accessed through the notebooks provided at https://github.com/zhou-1314/Garfield-reproducibility. These experiments were conducted on a high-performance computing infrastructure equipped with an NVIDIA A800-SXM4-80GB GPU, ensuring robust and efficient execution.

#### Datasets overview

The datasets presented in this manuscript can be broadly categorized into two groups: single-cell multi-omics datasets and spatial omics datasets. Unless otherwise specified, cell-type annotations and metadata were sourced directly from the original publications.

**Single-cell multi-omics datasets**

We collected major single-cell multi-omics sequencing datasets from a recent benchmarking study [71], comprising five single-cell RNA + protein datasets and nine single-cell RNA + ATAC datasets. These datasets were generated using a variety of sequencing technologies, including CITE-seq [1], SHARE-seq [4], SNARE-seq [3], ISSAAC-seq [5], and 10x Multiome (https://www.10xgenomics.com/products/). To ensure high-quality data, we employed Seurat V4’s quality control filters with parameters outlined in the original publications, effectively filtering out low-quality cells, RNAs in scRNA-seq data, and peaks in scATAC-seq data. Further details can be found in Supplementary Table S1, as well as the “Data availability” section.

**Spatial datasets**

*Slide-seqV2 mouse hippocampus*

The SlideSeqV2 dataset [6], representing the mouse hippocampus, was retrieved using the datasets.slideseqv2() function from Squidpy(v1.6.1). This dataset comprises a hippocampal puck with 41 786 observations at near-cellular resolution and 4000 genes. Since the dataset was preprocessed with log-transformed counts, we performed a reverse log normalization to derive the raw counts required for compatibility with Garfield. To facilitate benchmarking, we referenced the organizational annotations provided by the Allen Brain Atlas and manually annotated the dataset to generate ground truth for the experiment.

*Mouse olfactory bulb datasets*

We collected two MOB datasets, sequenced using Stereo-seq and Slide-seqV2, respectively. The Slide-seqV2 dataset comprises 20 139 observations at near-cellular resolution and 21 220 genes, while the subcellular MOB dataset from Stereo-seq contains 19 109 observations and 27 106 genes. These two datasets were subsequently merged into a single AnnData object, containing 39 248 observations and 18 279 genes. Batch effects arising from different sequencing platforms will be corrected during model training to ensure data alignment.

*High-resolution Xenium human breast cancer*

The 10x Xenium human breast cancer dataset and its associated metadata were obtained from 10x Genomics (https://www.10xgenomics.com/products/xenium-in-situ/preview-dataset-human-breast). This dataset comprises a total of 282 363 cells across two replicates: 164 000 cells in replicate 1 and 118 363 cells in replicate 2, with 313 genes probed. To ensure data quality, we filtered out cells with fewer than 10 total counts across all genes or with nonzero counts in fewer than three distinct genes.

*NanoString CosMx human non-small cell lung cancer*

The NanoString CosMx dataset for NSCLC [13] was sourced from https://nanostring.com/products/cosmx-spatial-molecular-imager/ffpe-dataset/nsclc-ffpe-dataset/. It encompasses eight tissue sections derived from five donors, with a total of 800 327 cells distributed as follows: 93 206, 97 487, and 91 691 cells for replicate 1, replicate 2, and replicate 3, respectively, of donor 1(lung5); 83 723 cells from donor 2(lung6); 77 391 and 115 676 cells for replicates 1 and 2 of donor 3(lung9), respectively; 66 489 cells from donor 4(lung12); and 76 536 cells from donor 5(lung13). Of these datasets, all but donor 5 (lung13) were employed to construct a detailed spatial transcriptomic atlas, providing a comprehensive representation of NSCLC. Conversely, donor 5 (lung13) was reserved as query data to assess Garfield’s capacity for seamless and rapid integration of newly acquired datasets, highlighting its adaptability and robustness.

To ensure data quality, cells with fewer than 50 total counts were excluded, yielding 766 313 high-quality cells. During model training, we accounted for variability by incorporating covariates such as sample identity and donor origin.

*Spatial ATAC-RNA multi-modal mouse brain*

The Spatial epigenome–transcriptome sequencing dataset for the mouse brain was obtained from Zhang *et al*. [72]. This dataset features a brain tissue sample from a juvenile (P22) mouse, comprising 9215 observations at spot-level resolution, with 22 914 genes and 121 068 peaks. To ensure high-quality data, we filtered out genes and peaks with counts detected in fewer than 46 cells (0.05% of all cells). Subsequently, we selected the 3000 most HVGs and 10 000 most HVPs, as identified by Scanpy. Finally, the two modality-specific AnnData objects were integrated into a single multimodal mdata object, which served as the input for Garfield’s analysis.

#### Experiment setup and baseline methods

**Benchmarking for single-cell multi-modal integration task**

In the single-cell multi-modal integration task, we systematically compared the performance of Garfield with four algorithms optimized for RNA and ATAC integration and another four designed for RNA and ADT integration. This benchmark study encompasses six state-of-the-art integrated algorithms. Similar to SeuratV4 and Multigrate, Garfield demonstrates versatility, enabling integration across different multimodal datasets (RNA + ATAC or RNA + ADT). The parameter configurations for these algorithms were determined based on the following guidelines:

*Garfield*. The final joint embeddings were obtained by making minimal parameter adjustments based on the specific single-cell multi-modal dataset. For RNA+ATAC datasets, the “sub_data_type” parameter was set to RNA+ATAC, and the genome parameter was configured as either human or mouse depending on the dataset’s species origin. For RNA+ADT datasets, only the “sub_data_type” parameter was modified to RNA+ADT. Across the paired single-cell integration datasets in this benchmark, all other parameters, including the loss weights, were kept at their default values to ensure a consistent configuration within this task family; how the loss weights are set across different task regimes (paired multi-omics, spatial, and multi-batch) is described above for Equation 37. A specific example can be found through https://garfield-bio.readthedocs.io/en/latest/tutorial/03.10x_pbmc_paired_scMulti_analysis.html.

*SeuratV4*. To perform single-cell multi-modal vertical integration, we adhered to the tutorial available on the Seurat(v4.1.0) website via https://satijalab.org/seurat/articles/weighted_nearest_neighbor_analysis.

*MultiVI*. Vertical integration using MultiVI(scvi-tools v1.2.0) was conducted in accordance with the instructions provided in the https://docs.scvi-tools.org/en/stable/tutorials/notebooks/multimodal/MultiVI_tutorial.html.

*MOFA+*. We integrated single-cell RNA expression and chromatin accessibility data following the guidelines outlined in the MOFA’s website(v1.9.2) at https://muon-tutorials.readthedocs.io/en/latest/single-cellrna-atac/pbmc10k/3-Multimodal-Omics-Data-Integration.html.

*totalVI*. We conducted the analysis based on the instructions from the https://docs.scvi-tools.org/en/stable/tutorials/notebooks/multimodal/cite_scrna_integration_w_totalVI.html. Key parameters for the analysis were set as latent_distribution = normal and n_layers_decoder = 2.

*scArches*. We followed the guidelines provided on the scArches(v0.6.1) website for RNA+ADT integration: https://scarches.readthedocs.io/en/latest/totalvi_surgery_pipeline.html. The model was trained with parameters “epochs = 200, plan_kwargs = dict.”

*Multigrate*. Single-cell multi-omics data integration was performed using the original Multigrate model(v0.0.2), as described in the https://multigrate.readthedocs.io/en/latest/notebooks/paired_integration_cite-seq.html. Specifically, we employed the “nb” loss function for RNA data and the “mse” function for ATAC and ADT data.

**Benchmarking for spatial niches discovery task**

We compared Garfield(v1.0.0) with three other spatial transcriptome algorithms: NicheCompass (v0.2.1), CellCharter (v0.3.2), and GraphST (v1.1.1). For this benchmark study, we selected mouse hippocampus data, characterized by its well-defined tissue structure, as the data source. We conducted eight training runs for each method on this dataset, while varying the number of neighbors. The neighbor counts ranged from 4 to 16, incremented in steps of 4, with each configuration evaluated in two independent runs. Specifically, the parameter configurations for these competing algorithms were set as follows:

*Garfield*. Garfield models were trained using the Adam optimizer with an initial learning rate of 0.001. A learning rate scheduler was employed, reducing the learning rate by a factor of 0.1 after four epochs without improvement, while early stopping was applied with a patience of eight epochs. The “GAT” network was selected as the Garfield encoder, and the augment_type was set to “dropout” for training and generating cell embeddings, with all other parameters left at their default settings. Detailed training configurations and procedures are available in the https://garfield-bio.readthedocs.io/en/latest/tutorial/05.Garfield_spatial_niche_slideseqv2_mouse_hippocampus.html.

*NicheCompass*. We followed the guidelines provided in the https://nichecompass.readthedocs.io/en/latest/tutorials/notebooks/mouse_cns_single_sample.html for model training, using the default hyperparameters.

*CellCharter*. As described in the https://cellcharter.readthedocs.io/en/latest/notebooks/codex_mouse_spleen.html, we utilized scVI (v1.2.1) for dimensionality reduction and data integration. CellCharter(v0.3.2) was then applied with default hyperparameters to extract latent representations.

*GraphST*. We adhered to https://deepst-tutorials.readthedocs.io/en/latest for model training, employing the default hyperparameters provided for GraphST(v1.1.1).

#### Benchmark metrics

**Single-cell integration task**

For comprehensive benchmarking of single-cell multi-omics integration, we employed ARI [73], NMI [74], cASW [75], and cLISI [76] as key metrics. ARI and NMI were used as primary measures to evaluate the concordance between known cell-type labels and the clusters identified by the Leiden algorithm. To ensure fair comparisons across algorithms, we automatically determined an appropriate resolution for each algorithm based on the number of original cell types, ensuring a consistent number of clusters.

The ASW metric assessed the precision of cell–cell distances calculated by each integration algorithm. Specifically, we used cell-type label-based ASW (cASW) to evaluate each algorithm’s ability to preserve biological variation. A higher cASW indicates better cell-type separation, reflecting improved accuracy in grouping cells within clusters (intra-cluster similarity) while maintaining distinctiveness from other clusters (inter-cluster dissimilarity).

We also utilized LISI to assess cell-type separation, denoting it as cLISI in our study. A lower cLISI value indicates more effective cell-type separation and greater conservation of biological variation. To ensure consistent interpretation of results, we applied linear transformations [77] so that higher cLISI values represent improved performance.

Finally, we introduced the biological variation conservation score, a total metric for evaluating integration algorithms. This score is calculated as the mean of ARI, NMI, cASW, and cLISI, providing a comprehensive measure of each algorithm’s ability to preserve biological variation across datasets.

**Spatial integration task**

For comprehensive benchmarking of spatial data integration, we employed ARI [73], NMI [74], AMI, HOM [78], ASW [75] as key metrics. These metrics were computed using the scikit-learn [79] or scIB [77] packages. To mitigate potential biases introduced by the resolution parameter in the Leiden algorithm, we performed Leiden clustering across a range of resolutions, from 0.1 to 2.0 in increments of 0.1, using the latent embedding from each algorithm. The performance of each algorithm was then evaluated based on the highest ARI and NMI values achieved across these resolutions.

*Adjusted Rand Index (ARI)*

The ARI quantifies the agreement between clustering results and ground truth labels by comparing pairwise groupings. Let *C* denote the ground truth labels and *K* the predicted labels. Define:

-
*m* : the number of sample pairs assigned to the same group in both *C* and *K*.
-
*n* : the number of sample pairs assigned to different groups in both *C* and *K*.

The unadjusted Rand Index (RI) is calculated as


\begin{align*} & RI = \frac{m + n}{\binom{n_{\mathrm{samples}}}{2}}, \end{align*}

where $\choose{n_{\mathrm{samples}}}{2}$ represents the total possible sample pairs.

Since RI is sensitive to random label assignments, particularly with similar numbers of clusters and samples, ARI adjusts for chance agreement by incorporating the expected value *E[RI]*. The adjusted formula is


\begin{align*} & ARI = \frac{RI - E[RI]}{\max(RI) - E[RI]} \end{align*}

This adjustment ensures ARI scores close to zero for random clusterings, providing a robust and normalized measure for evaluating clustering performance.

*Normalized mutual information*

NMI is s metric designed to quantify the alignment between predicted labels and the true labels. Given two sets of labels, *U* and *V*, representing the same *N* objects, the entropy of each set reflects the uncertainty associated with its partitions.

The entropy of *U* can be expressed as


\begin{align*} & H(U) = -\sum_{i=1}^{|U|} P(i) \log(P(i)), \end{align*}

where *P(i)* denotes the probability of randomly selecting an object from *U* that belongs to partition $U_{i}$, calculated as


\begin{align*} & P(i) = \frac{|U_{i}|}{N}. \end{align*}

Similarly, the entropy of *V* is computed as


\begin{align*} & H(V) = -\sum_{j=1}^{|V|} P^{\prime}(j) \log(P^{\prime}(j)), \end{align*}

with $P^{\prime}(j)$ representing the probability of an object being assigned to partition $V_{j}$, defined as


\begin{align*} & P^{\prime}(j) = \frac{|V_{j}|}{N}. \end{align*}

To measure the shared information between *U* and *V*, the MI is defined as


\begin{align*} & MI(U, V) = \sum_{i=1}^{|U|} \sum_{j=1}^{|V|} P(i, j) \log\left(\frac{P(i, j)}{P(i) P^{\prime}(j)}\right)\!, \end{align*}

where *P(i, j)* is the joint probability that an object belongs to both partition $U_{i}$ in *U* and $V_{j}$ in *V*, given by


\begin{align*} & P(i, j) = \frac{|U_{i} \cap V_{j}|}{N}. \end{align*}

The NMI provides a scale-invariant comparison by dividing MI by the average entropy of *U* and *V*, as follows:


\begin{align*} & NMI(U, V) = \frac{MI(U, V)}{\mathrm{mean}(H(U), H(V))}. \end{align*}

This normalization ensures that the score remains within a consistent range, facilitating comparisons across different datasets or clustering algorithms.

*Adjusted mutual information*

The AMI refines the MI by accounting for the expected similarity between two label assignments due to chance. It is defined as


\begin{align*} & AMI = \frac{MI - E[MI]}{\mathrm{mean}(H(U), H(V)) - E[MI]}, \end{align*}

where *E[MI]* represents the expected value of the MI under random labelings.

The expected MI, *E[MI(U, V)]*, is computed as


\begin{align*} & E[MI(U, V)] = \sum_{i=1}^{|U|} \sum_{j=1}^{|V|} \sum_{n_{ij} = (a_{i} + b_{j} - N)^{+}}^{\min(a_{i}, b_{j})} \frac{n_{ij}}{N} \log\left( \frac{N \cdot n_{ij}}{a_{i} b_{j}} \right) \end{align*}

\begin{align*} & \cdot \frac{a_{i}! \cdot b_{j}! \cdot (N - a_{i})! \cdot (N - b_{j})!}{N! \cdot n_{ij}! \cdot (a_{i} - n_{ij})! \cdot (b_{j} - n_{ij})! \cdot (N - a_{i} - b_{j} + n_{ij})!}, \end{align*}

where $a_{i} = |U_{i}|$ and $b_{j} = |V_{j}|$ denote the number of elements in partitions $U_{i}$ and $V_{j}$, respectively, and *N* is the total number of objects.

Here, $n_{ij}$ represents the number of shared elements between partition $U_{i}$ in *U* and $V_{j}$ in *V*. The term $(a_{i} + b_{j} - N)^{+}$ ensures nonnegative lower bounds for the summation.

By adjusting for *E[MI]*, the AMI eliminates bias caused by chance, ensuring a robust and normalized comparison of clustering results.

*Homogeneity (HOM)*

Homogeneity is a clustering evaluation metric that assesses whether each cluster consists exclusively of members from a single class. The principle behind this metric is that a well-defined cluster should contain only data points that are similar to each other.

The homogeneity score ranges from 0 to 1, where:

- A score of 1 indicates perfect homogeneity, meaning every cluster contains members from only one class.
- A score of 0 signifies complete heterogeneity, where clusters are composed of members from multiple classes.

This metric provides a measure of how effectively a clustering algorithm preserves class integrity within clusters.

*Average silhouette width*

The ASW metric evaluates the precision of cell–cell distances computed by each integration algorithm. In our study, niche label-based ASW was employed to assess each algorithm’s effectiveness in preserving niche-specific biological variation.

### Downstream analyses

**Data visualization**

To visualize the latent representations generated by various methods, we employed the UMAP algorithm to reduce the latent matrices to two dimensions. A k-nearest neighbor graph was first constructed using Scanpy’s pp.neighbors() function with default configurations, followed by dimensionality reduction via tl.umap(). The resulting 2D embeddings were then plotted through scanpy.pl.umap(). For spatial coordinate visualization, we used the scanpy.pl.embedding() function within the Scanpy(v1.9.1) package.

**Niche identification**

Leiden clustering is conducted on the model’s latent space via the scanpy.tl.leiden() function, defining each cluster as a niche. Relationships among niches are analyzed through hierarchical clustering using scanpy.tl.dendrogram(), with niche labels as input and “ward” or “single” linkage methods applied.

**Neighborhood composition analysis**

The neighborhood composition score evaluates the spatial proximity between identified niches and cell types. We utilized the built-in calc_neighbor_prop function from the Garfield package to compute this score. Specifically, Garfield firstly computes the nearest neighbors of each cell using spatial coordinates, identifies the cell types within the neighborhood, and calculates the proportion of each cell type among the neighbors.

**Spatial CHAOS score calculation**

To evaluate the spatial continuity and compactness of niches identified by Garfield, we computed the spatial CHAOS score [52]. For each slice *s*, a one-nearest-neighbor (1NN) graph was constructed for all spatial locations within each niche *m*. The edge weight between locations *i* and *j* was defined as


\begin{align*} & E_{ij} = \begin{cases} e_{ij}, & \mathrm{if}\ {i}\ \mathrm{and}\ {j}\ \text{are 1NN} \\ 0, & \mathrm{otherwise} \end{cases} \end{align*}

Here, $e_{ij}$ represents the Euclidean distance between *i* and *j*. The spatial CHAOS score for slice *s* was calculated as


\begin{align*} & \mathrm{CHAOS} = \frac{\sum_{m=1}^{M} \sum_{i,j \in m} E_{ij}}{N_{s}}, \end{align*}

where $N_{s}$ is the total number of spatial locations in slice *s*, *M* is the total number of spatial niches, and *i, j ∈ m* denotes that *i* and *j* belong to niche *m*.

**Enrichment score calculation for cell types**

To assess the enrichment of a specific cell type within a niche, we utilized an ”enrichment ratio” metric. This ratio compares the observed number of cells of the given type with the expected number, assuming a uniform distribution of that cell type across all niches. The expected cell count within the niche was calculated using the Chi-square test. An enrichment ratio exceeding 1, combined with a *P*-value below 0.05, indicates significant enrichment of the cell type in the niche.

**Proximal cell–cell co-location network construction**

To construct the proximal cell–cell interaction network, a spatial connectivity graph was created using the squidpy.gr.spatial_neighbors() function, with the interaction matrix computed via the squidpy.gr.interaction_matrix() function from the Squidpy(v1.6.1) package. The interaction strength $C_{ij}$ between cell types *i* and *j* was calculated as $C_{ij} = C_{ji} = \frac{X_{ij} + X_{ji}}{2}$, where $X_{ij}$ and $X_{ji}$ are the strengths of cell–cell interactions between cell types *i* and *j*.

To test statistical significance, a permutation-based test (default *n=1000*) was performed by shuffling cell-type labels while preserving the spatial connectivity graph. The *P*-value was determined as the proportion of permuted interaction strengths exceeding the true interaction strength. Cell types with fewer than 10 cells were excluded from the analysis.

**Differential expression gene analysis**

After obtaining the niches labels, differential expressed gene (DEG) analysis was performed on the identified niches using built-in calc_niches_marker function from Garfield package to identify DEGs.

**Functional enrichment analysis**

After obtaining the DEGs via differential expression gene analysis, functional enrichment analysis was performed using built-in calc_niches_enrich function from Garfield package to explore functional terms of each niche.

**Cell–cell interaction analysis**

CellChat V2 [55](v2.1.0) was employed to infer, analyze, and visualize intercellular communication between niches in spatial Xenium human BC data. Ligand–receptor interactions were analyzed using “Scanpy” normalized expression matrices, with interaction pairs sourced from CellChatDB, a curated database of mouse and human ligand–receptor interactions. Over-expressed ligands and receptors were identified for each niche, and communication probabilities were inferred by evaluating all ligand–receptor interactions linked to each signaling pathway.

**Key Points**

- Garfield provides a unified graph-based contrastive learning framework for integrating single-cell and spatial multi-omics data across transcriptomic, epigenomic, and proteomic modalities.
- Garfield preserves molecular specificity and spatial topology, enabling robust cell embedding, batch correction, spatial niche discovery, and query-to-reference mapping.
- Benchmarking across single-cell multi-omics and spatial omics datasets shows that Garfield matches or outperforms state-of-the-art integration and niche detection methods.
- Garfield enables cross-platform spatial niche transfer, supporting the construction and querying of spatial reference atlases across sequencing technologies.
- Applications to non-small cell lung cancer and breast cancer reveal heterogeneous tumor microenvironment niches and spatially organized immune interaction programs.

## Supplementary Material

Supplementary-material_bbag432

## Data Availability

The datasets analyzed in this study are all from publicly available datasets (Supplementary Table 1). Specifically, for single-cell multi-omics datasets, they were obtained from a recent benchmarking study [71], comprising five single-cell RNA + protein datasets and nine single-cell RNA + ATAC datasets. For spatial sequencing datasets, the SlideSeqV2 dataset, representing the mouse hippocampus, was retrieved using the datasets.slideseqv2() function from Squidpy. The mouse olfactory bulb tissue data generated by Stereoseq and Slide-seqV2 platforms can be accessed from https://github.com/JinmiaoChenLab/SEDR_analyses and https://singlecell.broadinstitute.org/single_cell/study/SCP815, respectively. The 10x Xenium human breast cancer dataset and its associated metadata were obtained from 10x Genomics (https://www.10xgenomics.com/products/xenium-in-situ/preview-dataset-human-breast). The NanoString CosMx dataset for NSCLC was sourced from https://nanostring.com/products/cosmx-spatial-molecular-imager/ffpe-dataset/nsclc-ffpe-dataset/. The Spatial epigenome–transcriptome sequencing mouse brain dataset was retrieved from https://www.ncbi.nlm.nih.gov/geo/query/acc.cgi?acc=GSE205055 (gene expression counts and spatial coordinates) and https://brain-spatial-omics.cells.ucsc.edu/ (peak counts and cell type labels). The details of all datasets used are available in the Methods. The annotation images from the Allen Mouse Brain Atlas can be accessed at https://mouse.brain-map.org/static/atlas. Garfield is available as a Python package, maintained at https://github.com/zhou-1314/Garfield. The Jupyter notebooks for reproducing our analyses and benchmarking results are available at https://github.com/zhou-1314/Garfield-reproducibility. Documentation is provided at https://garfield-bio.readthedocs.io/.
