# Supplementary material for: Graph-based contrastive learning enables unified integration and niche transfer across single-cell and spatial multi-omics
Source: Brief Bioinform. 2026 Aug 18;27(4):bbag432. doi: 10.1093/bib/bbag432 (PMC13481149; doi:10.1093/bib/bbag432)
Supplement: Supplementary file 1 [file supplementary-material_bbag432.zip › BIB-26-0923_R2_Supplementary_Information_bbag432.pdf]

Supplementary information

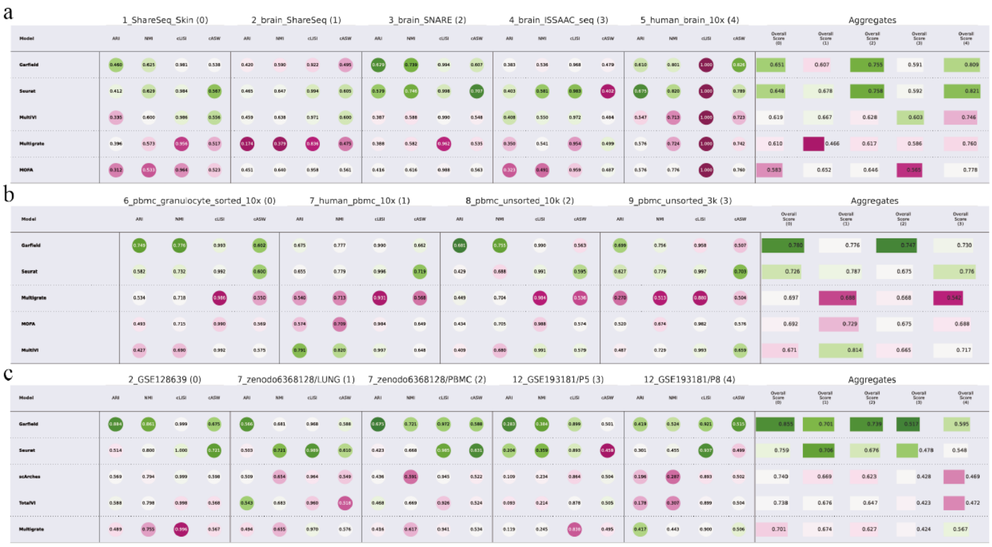

Fig. S1 Comparative Benchmarking of Garfield Across Diverse Single-Cell Multi-Omics Datasets. a. Garfield's performance is compared with Seurat V4, MultiVI, MOFA+, and Multigrate across five single-cell RNA+ATAC datasets. Metrics such as ARI, NMI, cLISI, and cASW demonstrate Garfield's superior clustering accuracy, biological variation preservation. **b.** Similar to panel a, but using datasets derived from the remaining 4 single-cell RNA+ATAC datasets. **c.** Garfield is assessed on four single-cell RNA+ADT datasets (GSE128639, Zenodo_LUNG, Zenodo_PBMC, and GSE193181), comparing its performance to Seurat V4, scArches, TotalVI, and Multigrate.

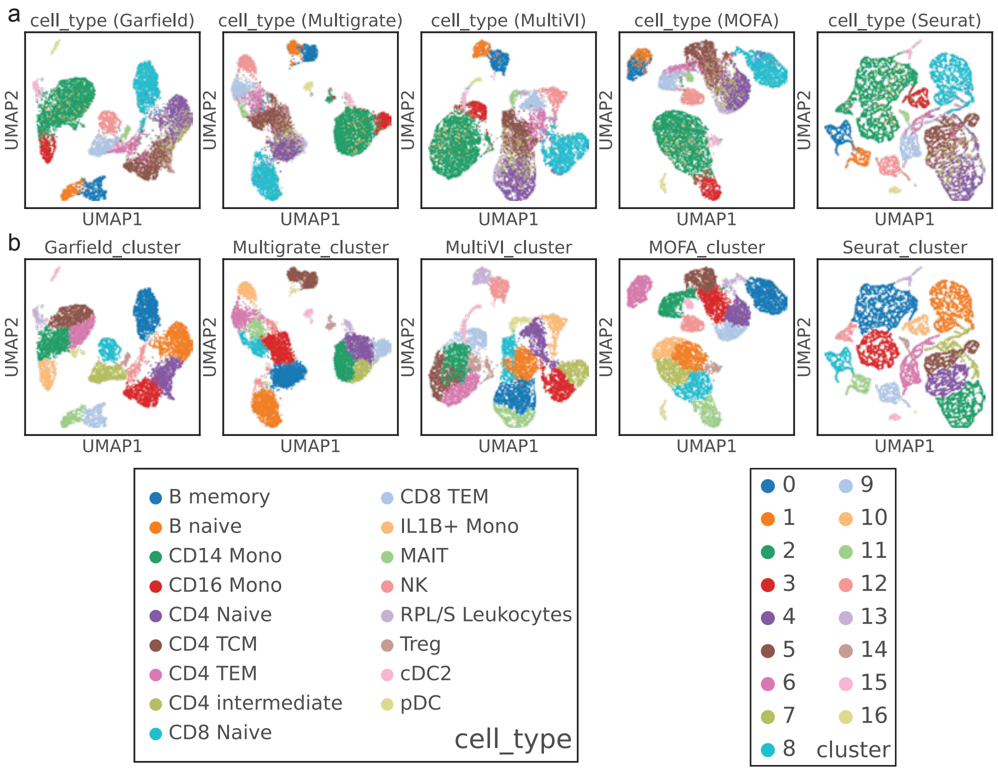

Fig. S2 Garfield extracts cross-modality representations from parallel single-cell RNA-ATAC datasets. To simplify the presentation, we focus on the results from one representative dataset (*pbmc_granulocyte_sorted_10x*), as the results from other datasets exhibit similar patterns. **a.** The UMAP visualization displays latent representations obtained through different methods, with points colored by the original cell type labels. **b.** The UMAP visualization highlights latent representations derived from different methods, with points colored by molecular clusters identified using the different approaches.

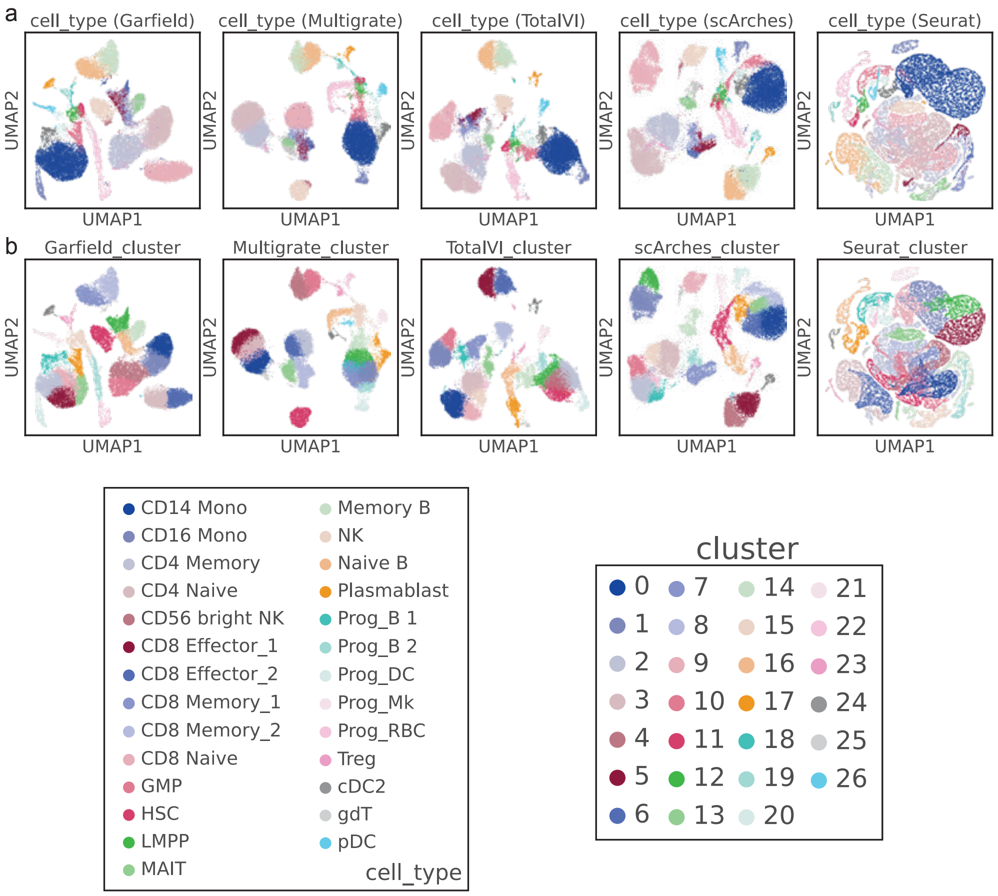

Fig. S3 Garfield extracts cross-modality representations from parallel single-cell RNA–ADT datasets. For clarity, only the results from one representative dataset (GSE128639) are presented, as the outcomes from other datasets are analogous. **a.** The UMAP visualizations of the latent representations—colored by the original cell type labels—obtained using various methods. **b.** The UMAP visualizations of the latent representations—colored according to molecular clusters identified via different approaches.

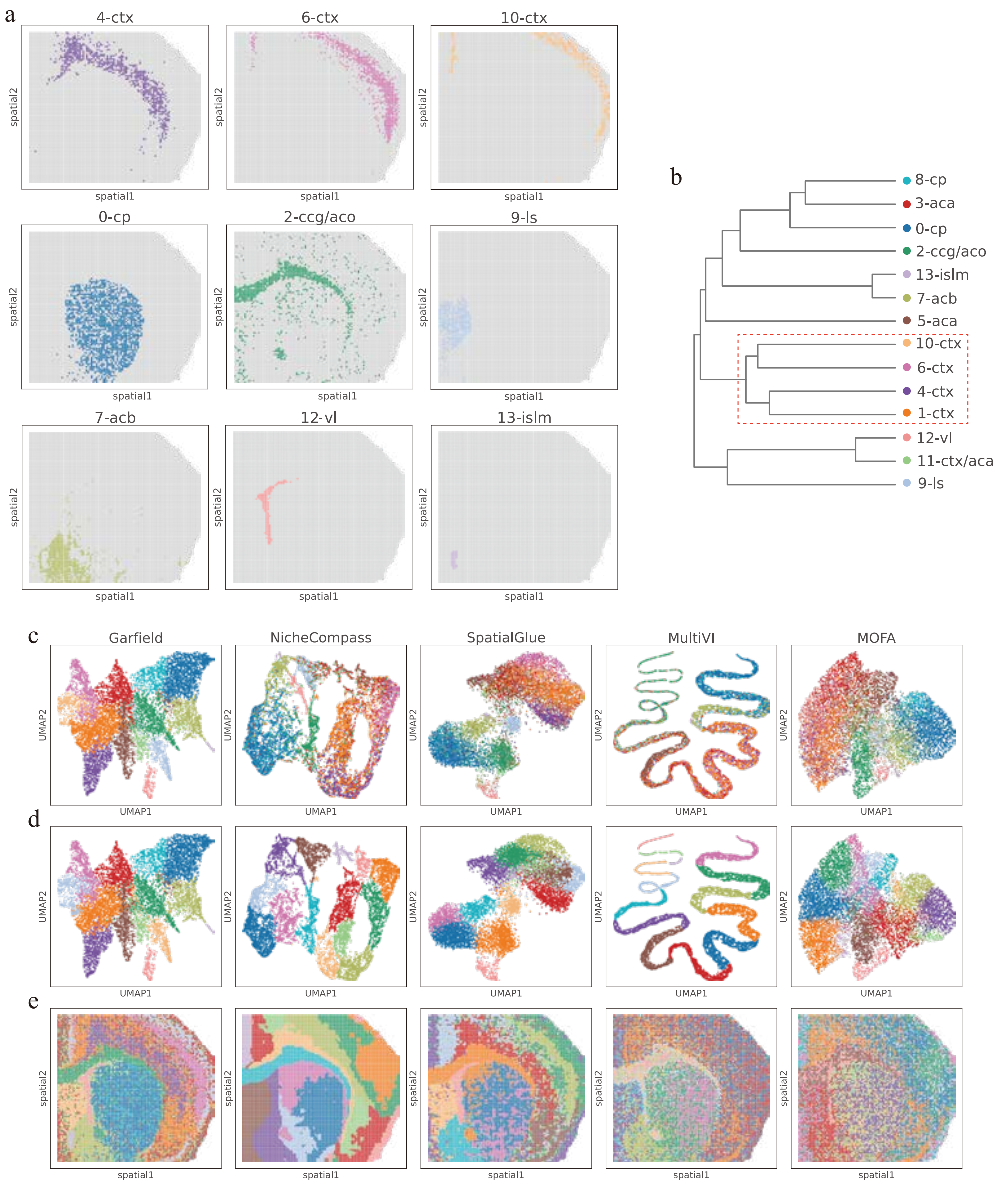

Fig. S4 Garfield enables high-resolution dissection of spatial epigenome-transcriptome landscapes in mouse brain samples. a. Spatial expression patterns of primary niches (e.g., ctx, cp, ls), and each niche is marked by its spatial coordinates. **b.** Dendrogram illustrating the hierarchical relationships between niches based on Garfield latent. **c-e.** UMAP and spatial maps comparing niche detection results across Garfield and alternative methods (NicheCompass, SpatialGlue, MultiVI, MOFA), which colored by ground truth niches labels(c) and niches identified based on different methods (d-e).

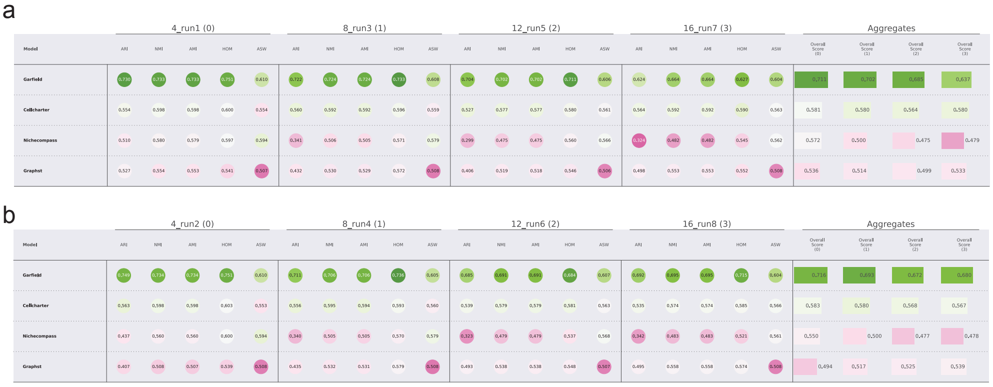

Fig. S5 Benchmarking Garfield and related algorithms with varying numbers of neighbors or seeds on a spatial multi-omics dataset. a. Garfield consistently achieves the highest scores across evaluation metrics, including ARI, NMI, AMI, HOM, and ASW, demonstrating its reliability in preserving clustering accuracy, spatial organization, and biological variation across different neighbor sizes. **b.** The benchmarking assesses performance variation caused by random seed differences. Garfield remains the most stable and accurate across all metrics, significantly outperforming competing methods. Its aggregate scores reinforce its robustness in generating reproducible results under different initialization conditions.

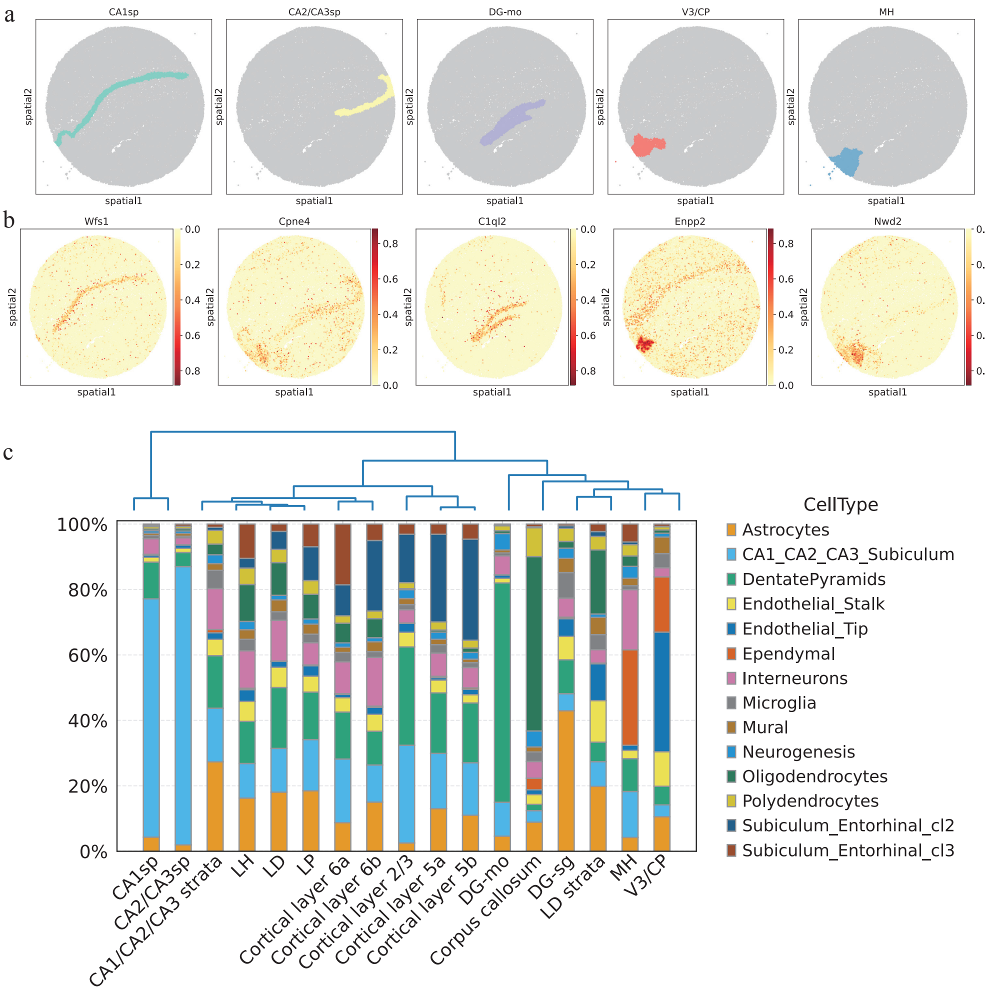

Fig. S6 Analysis of heterogeneity of hippocampal tissue using Garfield. a. Visualization of spatial niches corresponding to major anatomical regions, including CA1, CA2&CA3, DG (dentate gyrus), V3, and MH&LH (medial and lateral habenula). **b.** Spatial expression of region-specific marker genes, including Wfs1 (CA1), Cpne4 (CA2&CA3), C1ql2 (DG), Enpp2 (V3), and Nwd2 (MH&LH). **c.** Proportional representation of major cell types across hippocampal regions, including astrocytes, interneurons, oligodendrocytes, endothelial cells (stalk and tip), microglia, and dentate pyramids. Hierarchical clustering shows similarities in cellular composition among niches.

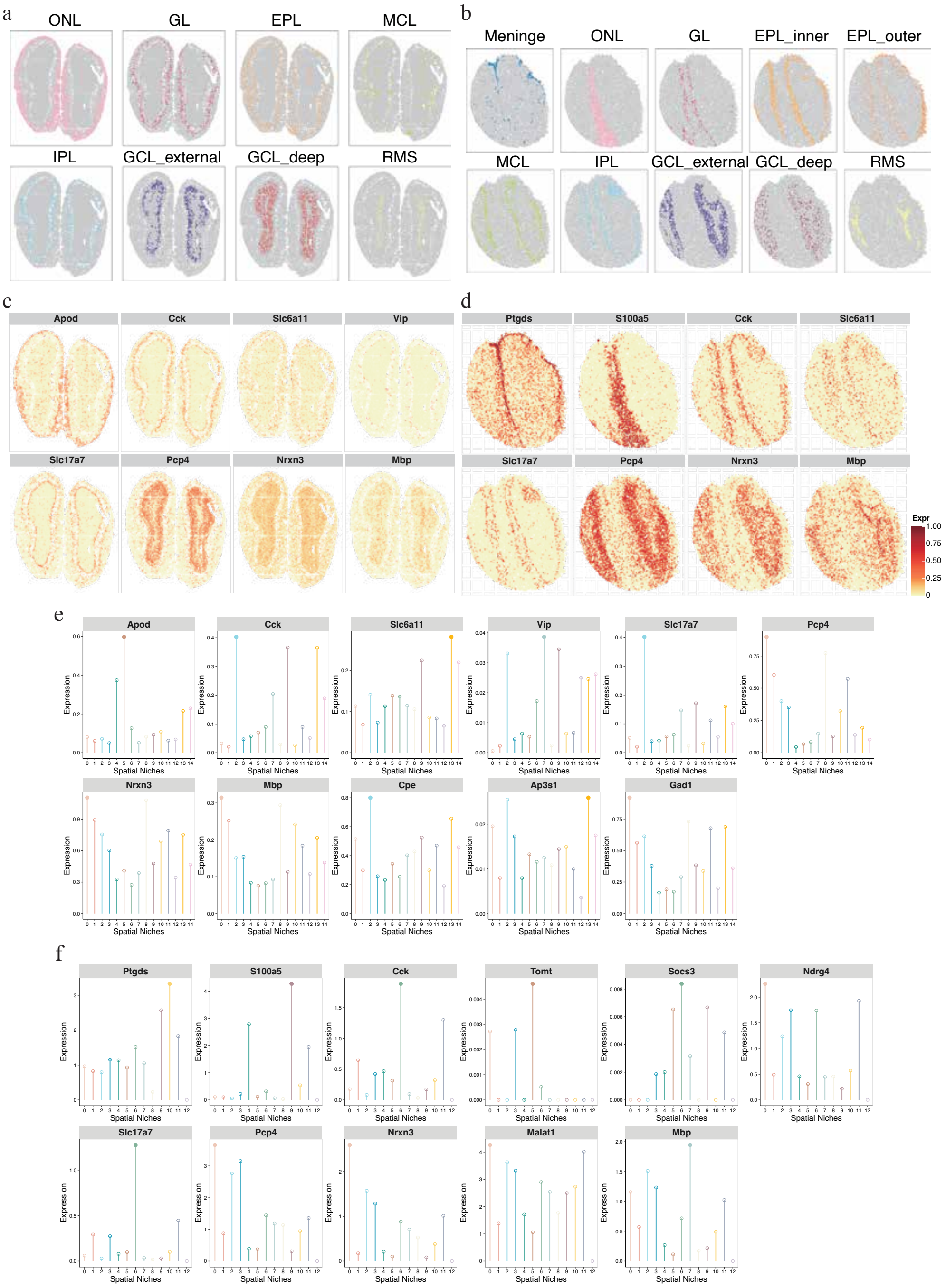

Fig. S7 Analysis of heterogeneity of mouse olfactory bulb tissue from different sequencing technologies using Garfield. a–b. Scatter plots showing the spatial distribution of key

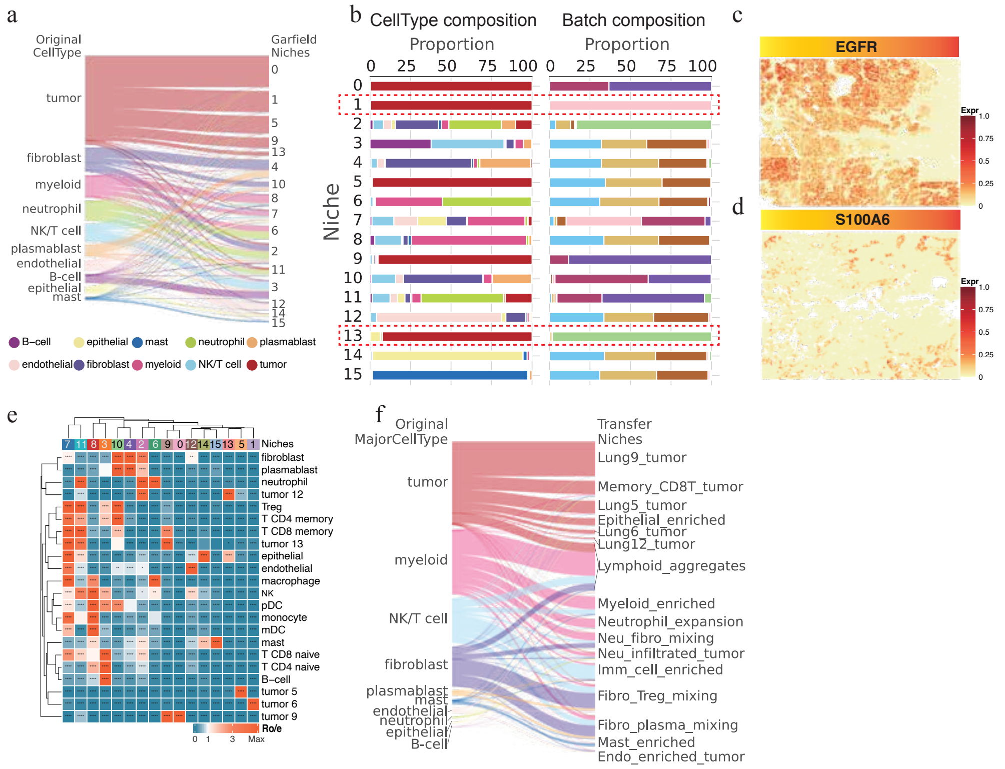

Fig. S8 Analysis of Garfield niches reveals cellular heterogeneity and spatial organization in the NSCLC tumor microenvironment. a. Mapping of major cell types to Garfield-defined niches, visualizing the distribution and contribution of cell types within each niche. **b.** Proportions of major cell types(left) or data source(right) in different niches. **c-d.** Spatial expression maps of two hub genes (EGFR and S100A6) for Niche 1 and Niche 13, showing their distinct and niche-specific enrichment patterns. **e.** Preferential localization of each cell type in different niches, based on the ratio of observed to expected cell numbers (Ro/e). A ratio of 1 is indicated by white, with values ¿1 signifying enrichment in the specific niche. **f.** Sankey plot illustrates how the original tumor cell map to the transferred niches.

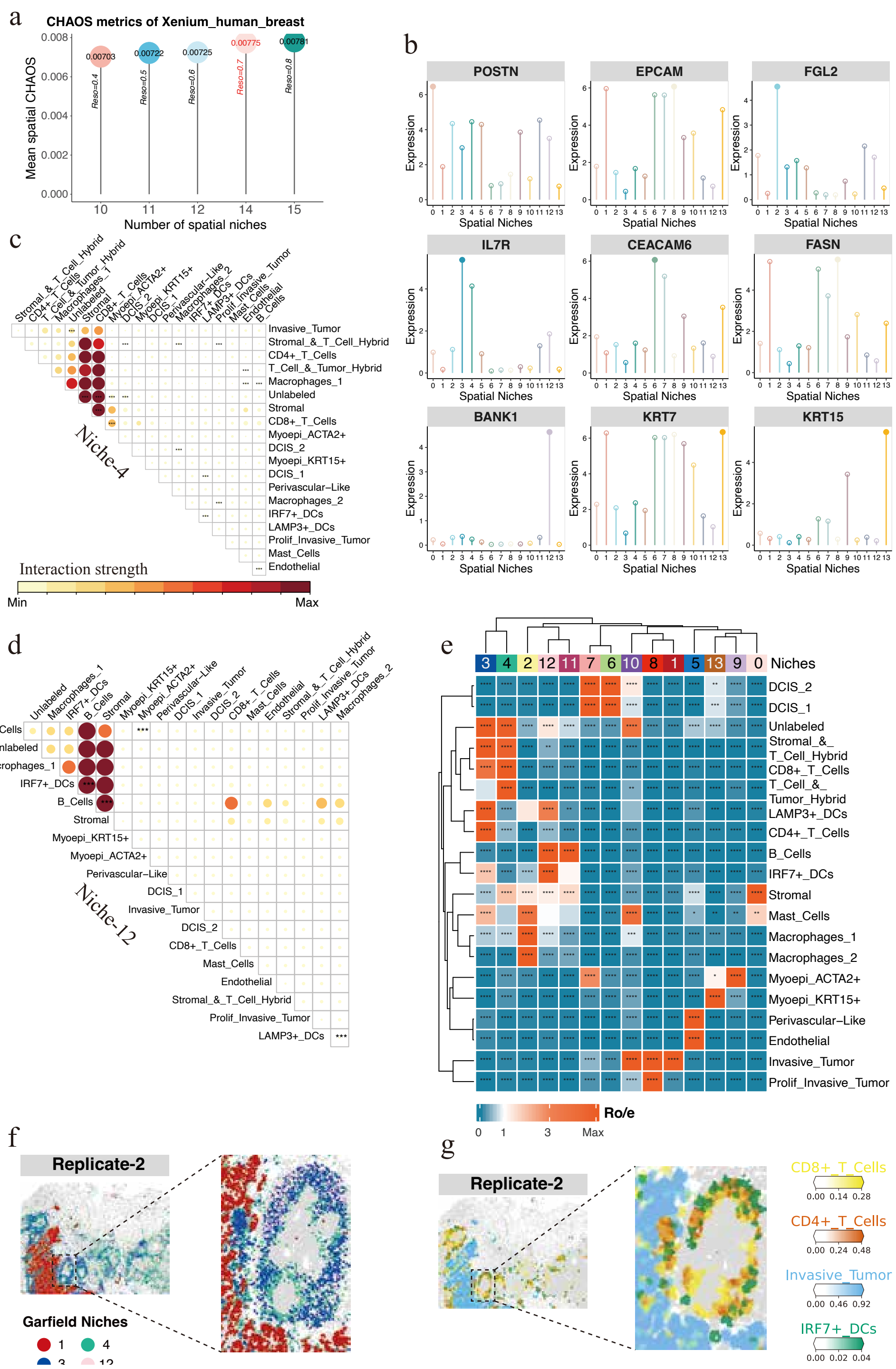

showing the mean expression of key marker genes (y-axis) across spatial niches identified by Garfield (x-axis), with solid circles marking the highest enrichment in each niche. **c-d.** Heatmaps illustrating proximal cell-cell interactions within niche 4 (c), and niche 12 (d), with the color scale indicating the interaction strength between cell types i and j. **e.** Preferential localization of spatial niches identified by Garfield, based on the ratio of observed to expected cell numbers (Ro/e). A ratio of 1 is indicated by white, with values ¿1 signifying enrichment in the specific niche. **f.** Spatial distribution of tumor-specific niche and lymphoid niches within replicate-2. The proximal local network at the tumor edge is highlighted. **g.** Spatial distribution of invasive tumor cells (yellow), CD8+ T cells (red), CD4+ T cells (blue) and IRF7+ DCs (green) within the replicate-2 tissue sections. Gray regions represent background areas.

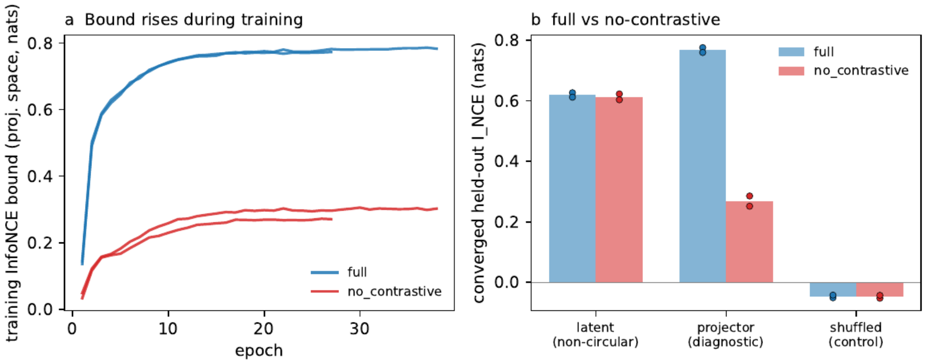

Fig. S10 Direct InfoNCE mutual-information bound between the two augmented views on Slide-seqV2 hippocampus (41,786 observations, two seeds). The instance contrastive loss is, by construction, an InfoNCE variational lower bound $I_{\mathrm{NCE}}(z_1; z_2) = \log K - \mathcal{L}_{(\mathrm{ins})}$ on the mutual information between the original and ApproxSVD-denoised node views (a fixed $K = 2 \times 256 - 1 = 511$ in-batch candidates, scored on the 256 seed anchors of each held-out validation batch, temperature 1); the bound is scored on a fixed held-out validation loader and is reported descriptively over two seeds as a variational lower-bound proxy, not as unbiased mutual information. **a**, The training-time bound, in the projection space on which $\mathcal{L}_{(\mathrm{ins})}$ acts, rises over epochs for the full model. **b**, Converged held-out bound (dots denote individual seeds). In the non-circular encoder-latent space the full and contrastive-disabled models are essentially equal (left), indicating that the feature/edge/adjacency reconstruction and KL objectives already render the two views' latents mutually informative; in the projection space the contrastive model attains a substantially higher bound (middle), an optimization diagnostic that is partly definitional because that projection head is updated only by the contrastive loss; a shuffled-positive-pair control collapses the estimate to near zero (right), confirming the estimator is meaningful.

Table 1 **Quantitative benchmark on the P22 spatial ATAC–RNA mouse-brain dataset (9,215 spots) against three external ground-truth annotations.** Each method's joint embedding is clustered with the scIB optimal-resolution Leiden protocol and scored by ARI against the original study's ATAC-derived clusters (14), RNA-derived clusters (11) and transferred cell types (33); the last two columns are the mean ARI and mean NMI across the three labels. Methods shown are deep-learning spatial/single-cell multi-omics integration models; the recent methods requested by the reviewers are SpaMosaic, SpaMI, soFusion and FGOT. The established matrix-factorization baseline MOFA+ (already benchmarked against Garfield in the main paper) is omitted from this recent/deep-learning comparison; for completeness, on this dataset MOFA+ attains a higher mean ARI (0.328) but a lower mean NMI (0.378) than Garfield. Among the deep-learning methods shown, Garfield (bold) attains the highest mean ARI and the tied-highest mean NMI, closely matched with SpatialGlue and ahead of all recent methods; values are single-run under each method's own recommended settings. FGOT† was run with locally reconstructed inputs (see text).

Method	ATAC ARI	RNA ARI	CellType ARI	Mean ARI	Mean NMI
**Garfield**	0.310	0.386	0.260	**0.319**	**0.391**
SpatialGlue	0.317	0.341	0.284	0.314	**0.391**
NicheCompass	0.229	0.342	0.221	0.264	0.340
SpaMosaic	0.253	0.295	0.210	0.253	0.369
SpaMI	0.177	0.287	0.220	0.228	0.337
soFusion	0.215	0.296	0.136	0.216	0.265
MultiVI	0.190	0.175	0.148	0.171	0.325
FGOT†	0.003	0.001	0.002	0.002	0.020

Table 2 Architectural and computational comparison of Garfield with recent spatial multi-omics integration methods. Methods marked "yes" in the last column are evaluated quantitatively on the P22 spatial ATAC–RNA mouse-brain dataset (Supplementary Table 1); the remaining methods are compared architecturally because environment or preprocessing constraints preclude a fair quantitative rerun (legacy TensorFlow 1.x and a precomputed gene–peak graph for MultiGATE; a two-slice alignment design with R/`mclust` dependencies for SpatialFuser; no provisioned environment and ATAC-untested tutorials for SpaOmicsVAE).

**Method**	**Integration mechanism**	**Batch alignment**	**Scalability**	**Quant.**
**Garfield**	Variational (VGAE); ApproxSVD-guided graph contrastive + reconstruction	Optional MMD (+DSBN)	Mini-batch; no dense $N \times N$; $>10^5$	ref.
SpaMosaic	Deterministic GAE; cross-modal graph contrastive + MNN bridge	MNN inter-batch graph	Mini-batch (>8000); sparse	yes
SpaMI	Deterministic GCN-AE; attention fusion + DGI contrastive + cosine align	None	Full-graph, dense $N \times N$; $\sim 10^4$	yes
soFusion	Deterministic GCN-AE (ZINB/Bernoulli decoders); self-attention + learned decoder fusion	None	Full-graph, dense $N \times N$; $\sim 10^4$	yes
FGOT	Optimal transport (non-neural); feature-guided unbalanced OT + alignment	OT coupling (no learned correction)	Mini-batch over clusters; dense $N \times M$	yes†
MultiGATE	Deterministic GATE (TF1.x); graph-attention + CLIP/InfoNCE cross-modal	None	Full-graph; $\sim 10^4$	no‡
SpatialFuser	Deterministic GAT transformer; cross-attention + differentiable Sinkhorn OT	OT/MNN cross-slice matching	Mixed; dense $N \times M$ at matching; $\sim 10^4$/slice	no‡
SpaOmicsVAE	Variational (VGAE); dual-graph cross-attention fusion	None	Full-graph; $\sim 10^4$	no‡

† FGOT's published P22 pipeline consumes Seurat-WNN preprocessed inputs that are not redistributed; we reconstructed the feature-selected matrices (HVG selection), the gene–peak feature graph (Ensembl GRCm38 TSS) and an approximate cross-modality cost in Python — a faithful-architecture reproduction with these approximations disclosed.

‡ Compared architecturally only (see caption). All methods support RNA, ATAC and (most) protein/spatial modalities.

Table 3 Runtime and peak GPU memory of Garfield across dataset scales. All values were measured on a single NVIDIA A800-SXM4-80GB GPU with the mini-batch settings reported in Methods (edge/node batch sizes; inductive neighbor sampling). Wall-time is the end-to-end training time. Peak memory is bounded by the mini-batch sampling rather than by the total number of observations, which is what allows Garfield to scale to atlas-size data: the full NSCLC atlas (∼700,000 observations, Fig. 4) trains in about two hours on a single GPU. "–" indicates peak memory was not logged for that run.

Dataset	#Observations	Epochs	Wall-time	Peak GPU mem
NSCLC (1% subset, multi-batch)	7,017	20	2.4 min (2.8 with MMD)	45.4 GB
10x Multiome mouse brain	10,647	50	14.6 min	67.9 GB
Slide-seqV2 hippocampus	41,786	50	48.0 min	–
NSCLC (full atlas)	702,199	100	124.7 min (∼2.1 h)	–

Table 4 Module-level ablation of Garfield's loss terms. Each term is removed in turn (all else fixed). 10x Multiome mouse brain (10,647 observations, real cell-type labels) and Slide-seqV2 hippocampus (41,786 observations, niche labels); mean±s.d. over two seeds {2024, 2025}. ASW = cluster average silhouette width; spatial coh. = fraction of spatial neighbours sharing the predicted cluster.

Variant	10x Multiome		Slide-seqV2 hippocampus		
	ARI	NMI	ASW	spatial coh.	niche ARI
Full model	0.483±0.039	0.605±0.002	0.220±0.014	0.804±0.038	0.451±0.032
− feature reconstruction	0.342±0.021	0.507±0.027	0.217±0.005	0.452±0.018	0.364±0.012
− cluster contrastive	0.402±0.034	0.580±0.001	0.212±0.023	0.775±0.078	0.442±0.031
− edge reconstruction	0.422±0.020	0.587±0.006	0.174±0.015	0.787±0.079	0.446±0.045
− contrastive (instance+cluster)	0.437±0.012	0.589±0.005	0.194±0.008	0.767±0.043	0.442±0.015
− clustering entropy $H(Y)$	0.440±0.107	0.590±0.014	0.196±0.006	0.706±0.125	0.436±0.021
− latent adjacency reconstruction	0.436±0.042	0.592±0.009	0.233±0.014	0.850±0.002	0.492±0.007
− instance loss	0.458±0.045	0.588±0.000	0.187±0.019	0.750±0.056	0.433±0.016

Table 5 Sensitivity to the message-passing neighbour-sampling parameter n_{sam} (= `num_neighbors`). 10x Multiome mouse brain (real cell-type labels) and a Slide-seqV2 hippocampus subset (3,735 observations, niche_type labels). Single seed (2024); peak GPU is for 10x. Default $n_{\text{sam}} = 3$.

n_{sam}	10x Multiome			Hippocampus subset	
	ARI	NMI	peak GPU (GB)	ARI	NMI
1	0.495	0.598	0.11	0.529	0.574
**3**	0.493	0.597	0.16	0.579	0.603
5	0.511	0.606	0.20	0.494	0.591
10	0.494	0.593	0.30	0.608	0.621
15	0.490	0.601	0.35	0.446	0.592

Table 6 **Sensitivity to the ApproxSVD rank q.** Dense 10x Multiome mouse brain (real cell-type labels) and an artificially sparsified Slide-seqV2 hippocampus stress test (298 observations, ≈65% read depth). Single seed (2024); default $q = 5$. On the small sparse subset the best-resolution Leiden partition is quantized, so ARI/NMI are nearly flat while the continuous embedding silhouette (ASW) varies smoothly with q.

q	**10x Multiome (dense)**		**Hippocampus (sparse, $n = 298$)**		
	ARI	NMI	ARI	NMI	ASW
1	0.457	0.594	0.657	0.749	0.2722
3	0.493	0.600	0.657	0.749	0.2725
**5**	0.480	0.598	0.657	0.749	0.2723
10	0.464	0.581	0.663	0.756	0.2725
20	0.495	0.597	0.657	0.749	0.2723

Table 7 **Local robustness to individual loss weights.** Each loss weight is scaled by ×0.1 and ×10 around its task setting on 10x Multiome mouse brain (real cell-type labels, single seed 2024); ARI is reported. The unperturbed baseline ARI is 0.486.

**Loss weight**	ARI (×0.1)	ARI (baseline)	ARI (×10)
λ_{edge} (edge recon.)	0.482	0.486	0.506
λ_{omics} (feature recon.)	0.496	0.486	0.483
λ_{adj} (latent adj. recon.)	0.519	0.486	0.512

Table 8 **MMD batch-alignment ablation.** NSCLC 1% multi-batch subset (7,017 observations, cell-type labels), **single seed (2024)**. Enabling MMD improves cell-type clustering and batch mixing (lower batch ASW and higher kNN batch entropy indicate better mixing).

**Setting**	cell-type ARI	NMI	batch ASW	kNN batch entropy
without MMD	0.151	0.360	0.205	0.145
with MMD	0.182	0.444	-0.035	0.226

Table 9 **Complementary label-level mutual-information readout on Slide-seqV2 hippocampus.** Mutual information (MI), normalized mutual information (NMI), and adjusted mutual information (AMI) are computed between Garfield-derived niche clusters and the 17-class reference niche annotations; mean±s.d. over two seeds {2024, 2025}. This downstream label readout is separate from the view–view InfoNCE bound in Supplementary Fig. S10 and does not isolate the contrastive objective.

**Variant**	**MI (nats)**	**NMI**	**AMI**
Full model	1.481±0.046	0.611±0.027	0.611±0.027
− feature reconstruction	1.295±0.025	0.485±0.004	0.485±0.004
− contrastive (instance+cluster)	1.513±0.025	0.610±0.017	0.610±0.017
